# Factors influencing immunogenicity and safety of SARS-CoV-2 vaccine in liver transplantation recipients: a systematic review and meta-analysis

**DOI:** 10.3389/fimmu.2023.1145081

**Published:** 2023-09-05

**Authors:** Xinyi Luo, Fabrice Yves Ndjana Lessomo, Zhimin Yu, Yong Xie

**Affiliations:** ^1^ Queen Mary School, Nanchang University, Nanchang, Jiangxi, China; ^2^ Department of Gastroenterology, the First Affiliated Hospital of Nanchang University, Nanchang, Jiangxi, China; ^3^ Department of Cardiology, Zhongnan Hospital, Wuhan University, Wuhan, Hubei, China

**Keywords:** liver transplant, SARS-CoV-2, adverse effect, Vaccine, Meta - analysis

## Abstract

**Background:**

This review summarizes the factors influencing the efficacy and safety of the COVID-19 vaccine in LTR through meta-analysis, hoping to provide strategies for vaccine use.

**Methods:**

Electronic databases were screened for studies on mRNA vaccines in LTR. The primary outcome was the pooled seroconversion rate, and the secondary outcome was the incidence of adverse events+breakthrough infections. Subgroup analyses were made based on BMI, associated comorbidities, presence of baseline leukopenia, time since transplant, and drugs used.

**Result:**

In total, 31 articles got included. The pooled seroconversion rate after at least two doses of SARS-CoV-2 vaccination was 72% (95% CI [0.52-0.91). With significant heterogeneity among studies I^2^ = 99.9%, the seroconversion rate was about 72% (95%CI [0.66-0.75]), from the studies reporting two doses of vaccine slightly higher around 75%(95%CI [0.29-1.22]) from studies reporting three doses. The pooled seroconversion rate within the lower to normal BMI group was 74% (95% CI [0.22-1.27], Pi=0.005) against 67% (95% CI [0.52-0.81], Pi=0.000) in the high BMI group. The pooled seroconversion rate in the ‘‘positive leukopenia’’ group was the lowest, 59%. Leukopenia could influence the vaccine seroconversion rate in LTR. From the time since transplant analysis after setting seven years as cut off point, the pooled seroconversion rate after at least two doses of COVID-19 vaccination was 53% (95% CI [0.18-0.83], P=0.003, I^2^ = 99.6%) in <7years group and 83% (95% CI [0.76-0.90], P=0.000 I^2^ = 95.7%) in > 7years group. The only time since transplantation had reached statistical significance to be considered a risk factor predictor of poor serological response (OR=1.27 95%CI [1.03-1.55], P=0.024). The breakthrough infection rate after vaccination was very low2% (95% CI 0.01-0.03, I^2^ = 63.0%), and the overall incidence of adverse events, which included mainly pain at the injection site and fatigue, was 18% (95%CI [0.11-0.25], I^2^ = 98.6%, Pi=0.000).

**Conclusion:**

The seroconversion rate in LTR vaccinated with at least two doses of mRNA COVID-19 vaccine could be significantly affected by the vaccine type, immunosuppressant used, BMI, leukopenia, associated comorbidities, and time since transplantation. Nevertheless, booster doses are still recommended for LTR.

## 1 Introduction

Coronavirus disease 19 (COVID-19), caused by severe acute respiratory syndrome coronavirus 2 (SARS-CoV-2), has infected nearly 600 million people worldwide and caused more than 6 million cumulative deaths, causing a significant global economic and medical burden. The development and application of SARS-CoV-2 vaccines are one of the most important measures to reduce the infection rate of SARS-CoV-2.

Several clinical trials have confirmed the efficacy of the SARS-CO V-2 vaccine, which is being used worldwide (1). However, these studies often excluded patients treated with immunosuppressive drugs, including LT patients (2–4).

In organ suppression or tissue transplantation, the transplant recipient’s immune system will produce an immune response to the transplanted organ of the transplant donor, so drugs are needed to suppress the excessive rejection of the immune system (5). This may have potential implications for the efficacy and safety of vaccination in these patients and may increase the risk of infection (6). Multiple questions have arisen regarding the effectiveness of vaccination against SARS-CoV-2 in LTR, including factors such as the different complications that occur in LT recipients, differences in the duration of LTation, and the use of different immunosuppressive agents (7).

Several studies have reported that the COVID-19 vaccine could work well in people who have had liver transplants, however most of the studies had either a sample sizes that were insufficient to predict outcomes accurately or didn’t include the analysis of other factors like BMI, type of comorbidities, etc. BMI is emerging as an important factor influencing the effectiveness of vaccines, evidences have suggested that high BMI levels were associated with impaired immune response leading to low vaccine response in setting of influenza, hepatitis and other vaccines. However, in the context of Covid 19 it should be noted that individual with obesity have been indexed as high risk group for severe outcomes, and preclinical data suggested that those peoples would generate a lower seroconversion rate compared to others. Nevertheless, the clinical significance of BMI in context of covid 19 vaccine among LTR still remain unelucidated. On the other side liver transplant recipients often experience leukopenia due to their immunosuppressive medication which will obviously affect their immune responses to infections. This raises concern about their ability to mount an effective immune response to COVID 19 vaccination. And therefore warrant the investigation of the impact of both leukopenia and drugs on the immunogenicity of Covid19 vaccine among LTR (8–10). Finally, studies have shown that comorbidities such as diabetes, hypertension, or heart disease may have a reduced immune response to the vaccine compared to those without underlying health conditions, but this still needs to be demonstrated in the setting of Covid-19 vaccinated LTR (11).

Up to days, the safety of the COVID-19 vaccine has remained a substantial matter of concern among LT recipients, their families, and their caretakers. Therefore, this review aims to summarize the factors that could potentially affect vaccine efficacy and safety in LT recipients and provide references for future studies.

## 2 Methods

This meta-analysis was conducted according to the panel recommendation of the Meta-Analysis of Observational Studies in Epidemiology (MOOSE) and the Preferred Reporting Items for Systematic Reviews and Meta-Analyses (PRISMA) guidelines (12).

### 2.1 Database search

A literature search was done using PubMed and Cochrane Library databases (the last search was on March 25^th^, 2023) using the keywords Liver Transplantation combined using the operator ‘AND’ COVID-19, SARS-COV-2 ‘AND’ Vaccine ‘OR’ Vaccination.

*((Liver transplantation) AND ((COVID-19) OR (SARS-COV-2))) AND ((vaccine) OR (vaccination))*

All titles and abstracts were screened to collect studies that may be relevant to LT patients and SARS-COV-2. References for the included studies were manually searched to identify other relevant studies. Titles and abstracts were reviewed, followed by full-text screening, and quality assessment.

### 2.2 Inclusion and exclusion criteria

Specific inclusion criteria for the systematic review and meta-analysis were as follows: (1) describe seroconversion of LT recipients with the second, or booster doses of the messenger RNA-based Sars-Cov-2 vaccine; (2) Study reporting on novel coronavirus infection or death in LT recipients after vaccination; (3) the occurrence of local or systemic adverse reactions after vaccination in LT recipients; (4) Studies including at least 10 LT recipients only were considered to avoid significant bias caused by small sample size. The analysis excluded case reports, case series (< 10 cases), guidelines, surveys, and editorial reviews.

### 2.3 Data extraction

From each eligible study the following data were extracted by one author and reviewed by a second author these include: information on the authors of published literature, study population, patient characteristics (drugs, complications, BMI, comorbidities, etiologies of transplantation, comorbidities and presence of leucopenia or not), the time since liver transplantation, type and number of doses of mRNA vaccine BNT162b2 or mRNA-1273 received, post-vaccination infection rates, and presence of not of adverse events.

### 2.4 Outcome indicators

A single-arm meta-analysis (pooled data analysis) was performed in absence of control arms. All studies on the efficacy and safety of the Novel Coronavirus vaccine in LTR were evaluated. Since seroconversion and breakthrough infection are considered markers of the effectiveness of the SARS-CoV-2 vaccine, the primary outcomes was the rate of seroconversion and the occurrence of novel coronavirus infection following second, and booster doses of the SARS-CoV-2 vaccine administration in LTR. the secondary outcome was the occurrence of adverse events after vaccination in LTR.

Subgroup analyses were also performed to assess the effect of others factors(predominant comorbidities, BMI, presence of leukopenia, time from transplant to vaccination, effects of different drugs on the seroconversion rates in LT recipients). The comorbidities were categorized as cardiovascular (hypertension, coronary disease etc.), endocrine (diabetes mellitus), respiratory (COPD, respiratory failure etc.), renal (CKD etc.). to assess the role of BMI, Studies reporting many cases of obesity or of BMI> 25 were labelled as High BMI and those with BMI lower than 25 or few cases of obesity were labeled as low to normal BMI. As for leukopenia all the studies reporting on either the mean of white blood cell count or the presence of leukopenia in included cohorts were grouped in two categories that were no leukopenia and positive leukopenia.

### 2.5 Study characteristics and quality assessment

Because most of the included studies were observational prospective or retrospective investigations, the quality of each study was independently assessed by two investigators (N.F, X.L) using the Newcastle-Ottawa Scale (NOS) and inconsistencies were resolved by consensus.

### 2.6 Data analysis

The analyses were conducted using the Stata software 13 MP (StataCorp, College Station, TX). A random-effects model was chosen because of substantial heterogeneity accross the included studies. The pooled rates of adverse events were computed under the random-effects model and the inverse variance method. The heterogeneity was determined by the I^2^ and P value of heterogeneity>0.1.

### 2.7 Publication of bias assessment

The funnel plot asymmetry was used to confirm the existence of publication bias across the studies. Which was confirmed by Egger’s test.

### 2.8 Sensitivity analyses

Sensitivity analyses were generated from the Stata software *via* the ‘‘studies omission approach’; when the omission of selected studies didn’t affect much the pooled results; then, the results was to be considered valid.

## 3 Results

**Figure 1:**
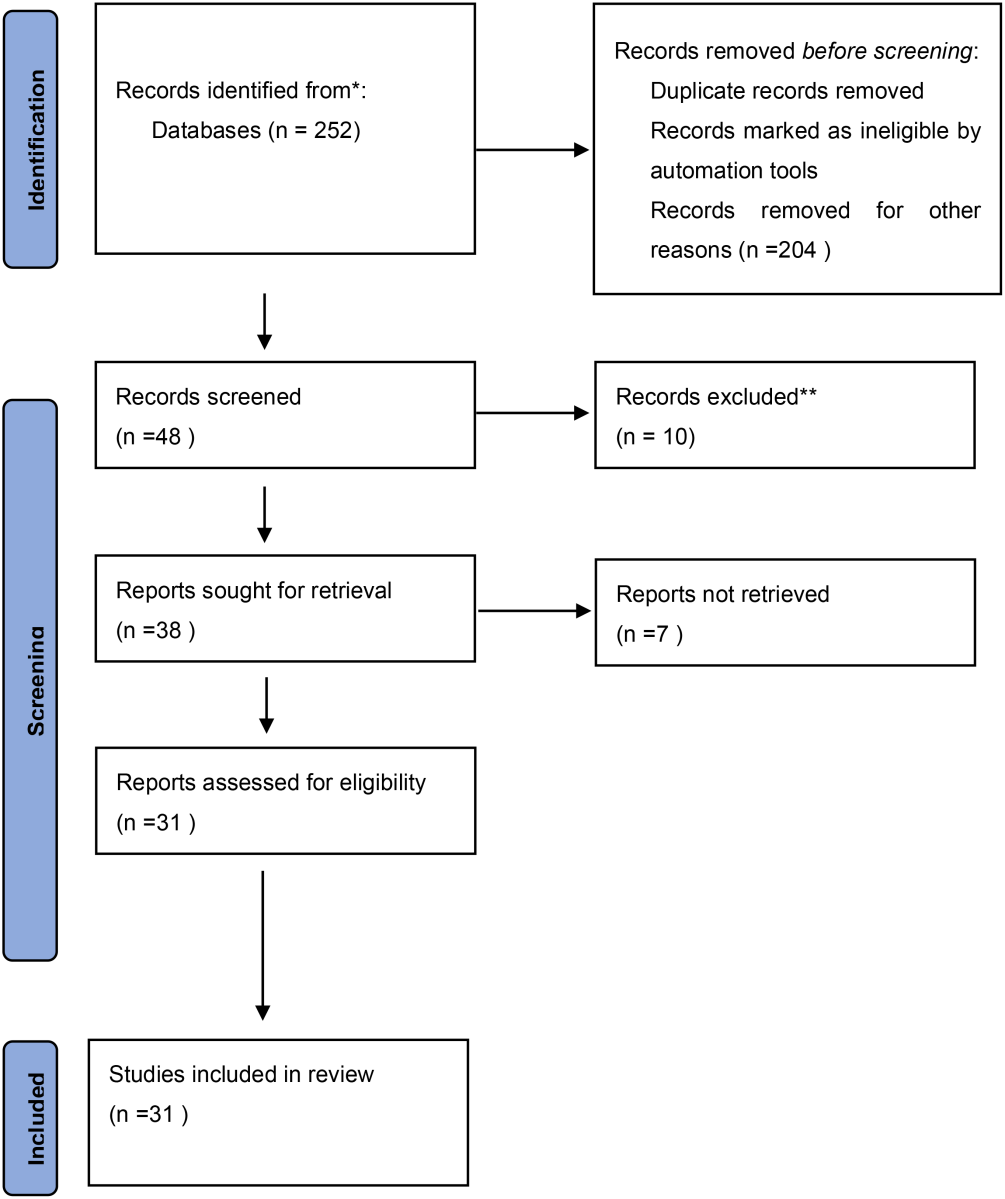
PRISMA 2020 flow diagram for new systematic reviews which included searches of databases and registers only.

A total of 252 records were identified through an initial keyword search. After screening by title and abstract, 208 articles were removed for multiples reasons, including articles that did not report outcomes of interest, review articles, and articles not in English. This left 44 articles for further full-text screening, among which only 30 studies were included in our analysis. The PRISMA flow diagram gives a detailed description of the selection process (**Figure 1**). The baseline characteristics of included studies are shown in **Tables 1**, **2** (13–18, 20–26, 28–42, 44–50).

**Table 1:** characteristic of included studies describing the effectiveness and safety of SARS-CoV-2 vaccines in LTR patients.

Study	year	type	age	male	Time after LT, years	Etiology of LT	predominant comorbidities and presence of leukopenia or not, BMI	post2	total2	post3	total3	Type of vaccine	Type of antibody	Assay of antibody testing	Immunosuppressive agents	univariate or multivariate analysis for negative serology	AE post vaccination
**SEBASTIAN** (13)	2022	Retrospective cohort	55 (46–63)	0.56	5.1 (1.8–9.7)		CVD 145 (36.2%)	8	45	3	454	BNT162b2			Mycophenolate, Azathioprine, no antimetabolic tacrolimus, erolimus	Multivariate, logistic regression	
							RESP 42 (10.5%)										
							DM 89 (22.2%)										
**Pierluigi** (14)	2022	retrospective	57.9 (51.8-62.8)	92 (70.2)	94 (49-189)month	HCV 28	DM 46	123	131			BNT162b2	anti-SARS-CoV-2 s-RBD IgG antibodies	ELISA	Tacrolimus cyclosporine	univariate	modest pain in the vaccination injection site 61
						HBV 21	HTN 58								MMF EVEROLIMUS Prednisone		fever, asthenia or myalgia 7
						NASH 0	Dyslipi 29										
						AH 58	BMI 26										
						AI 13	Leu 5.649										
						Others 11	Neu 3.357										
							Asc 4										
**Chombchanat** (15)	2022	prospective	14.5 ± 1.8	8 (66)	102 (58–160) months	Biliary atresia 7 (58.4)	BMI, kg/m2 (mean ± SD) 18.5 ± 2.8	95%	12	NA		BNT162b2	anti-SARS-CoV-2 antibody NAbs against SARS-CoV-2	Elecsys® anti-SARS-CoV-2 S, Svnt and ELISA assay,	Corticosteroid 3 (25) Cyclosporin 6 (50) Tacrolimus 6 (50)		PAIS 60%
						Autoimmune liver disease 1 (8.3)									No of immunosuppressant 2–3; 6(50 )		F&Chi 21%
						Alagille syndrome 1 (8.3)											Headache 18%
						Others 3 (25)											Myalgia 25%
																	Diarrhea 5%
**Ericka** (16)	2022	Prospective	58 [49–67]	40 (30.5%)			Coronary artery disease 7 (5.3%)	100	119			BNT162b2 (Pfizer-BioNTech)/mRNA	IgG anti-SARS-CoV-2	ELISA	MMF 96		No AE
															Calcineurin inhibitors 98		
															No of immunos 2-3, 97		
**Anna (** 17)	2002	retrospective	56 (42–65)	46 (46.9)	7 (3–13.3)	Alcoholic liver disease 14 (14.3) Autoimmune 25 (25.5)	24 (21.5–26.9)	11	12	56	59	BNT162b2 (Pfizer-BioNTech)/mRNA	anti-NC-SARS-CoV-2 Ig	Elecsys SARS-CoV-2 S assay	Tacrolimus 17 (17.3)		16 cases of breakthrough infection after one year of vaccination
						Viral 15 (15.3	Diabetes 32 (32.7)								Cyclosporine 3 (3.1)		
						Hepatocellular carcinoma 8 (8.2)	Arterial Hypertension 52 Leucocytes 5.6 (4.1–7.3)										
**Chang** (18)	2022		60 IQR: (46–67)	0.56	6 IQR:			30 day:136	161			BNT162b2 (47%) mRNA-	anti-S1		mycophenolate: 37/50		
					(3–16)							1273 (53%)					
**Balsby** (19)	2022	Prospective Cohort				NA	NA					BNT162b2 (Pfizer–BioNTech)	anti-spike S1 IgG response				
**Cholankeril** (20)	2021	prospective observational study	IQR: 63 (51-68)	0.7	3.3 (1.7-8.3)	ALD 24 (35)	Obesity* 37 (54)	33	69			BNT162b2(100%)	anti-spike S1 IgG		Tacrolimus	Multivariate model	
						NASH 13 (19)	DM 33 (4										
						HCC 21 (30)	Chronic kidney disease stage III/IV‡ 36 (52)										
							Leucopenia§ 9 (13										
**John** (21)	2022		IQR: 68.9 (7.9)	0.976	IQR: 5.6 (5.0)	NA	NA		1133			BNT162b2 mRNA vaccine					Breakthrough: 24 of 1133 vaccinated and 43 of 791 control subjects, symptomatic COVID-19 in 14 vaccinated and 26 control subjects, and COVID-19–related death in 2 vaccinated and 11 control subjects.
**Cuadrado** (22)	2022	retrospective cohort	63 (IQR, 56–68)	0.767	7.0 years (IQR, 4–12)	Alcohol 56 (43.4)	Hypertension 78 (60.5)	113	129			mRNA-1273	anti-spike IgG		CNIs	logistic regression	
						HCV 24 (18.6)	Diabetes 47 (36.4)										
						Other 37 (28.7)	Chronic kidney disease 46 (35.9)										
						Hepatocellular carcinoma 52 (40.3	Chronic lung disease 12 (9.3)										
**D’Offizi** (23)	2021	NA	59 (IQR 56-61)		6 years (IQR 3-10,			47	61			BNT162b2	anti-spike IgG		CNIs, MMF or steroids	Multivariate regression analysis	
					range 1-26)												
**Davidov** (24)	2022	prospective	65 (IQR 52-70)	0.574	7 years (IQR 4-18)		DM 24			60	61	BNT162b2	anti-spike IgG	ELISA			adverse effects:37%, 29%:localadverse reactions (mostly localized post-injection pain) and 22% reported systemic adverse reaction (mostly fatigue)
							HTN 28										
							CKD 39										
							mBMI=25						neutralizing antibody	neutralization assay			
							Leu 5.6						T-cell immune response	ELISpot assays			
**Davidov** (25)	2022	prospective	59 ± 15 (Mean+SD)	0.566	7 years (IQR,4-16)	NASH 13	Diabetes mellitus 31	55	76			BNT162b2	anti-spike IgG		Tacrolimus	logistic regression analysis	
						Viral hepatitis 27	Hypertension 36										
						Others 23	Chronic kidney disease 25										
							BMI 25										
							Leu K/μL 6.0										
** **													neutralizing antibodies		Everolimus		
** **															Prednisone		
** **															MMF		Local AE: 19.7%; Systemic AE:19.7%
** **															Double immunosuppression		
** **															Triple immunosuppression		
**Fernández-Ruiz** (26)	2021		NA	NA	NA		Htn 32	7	13			mRNA-1273 (100%)	Anti-spike Ab	ELISA	Tacrolimus		overall: 27.3%: pain at the injection site (n=6), headache (n=3), fatigue (n=2), fever (n=1), tachycardia (n=1), and nausea (n=1).
							DM 11										
							COPD 4										
							Obesity 6										
** **															MMF/MPS		
** **															mTOR inhibitor		
** **															Prednisone		
**Furian** (27)	2022	prospective	64.0 ± 7.7	0.74	141.4 ± 204.9 months			45	50				anti-spike				
													(S), S1, S2, RBD, and nucleocapsid IgG				
**Giannella** (28)	2022	prospective	NA	NA	NA			144	182			BNT162b2	anti-spike RBD Ab	ECLIA			
** **												mRNA-1273					
**Harberts (** 29)	2022	prospective	59.0 (51.0–68.3)	0.604	NA					97	106	BNT162b2	anti-S RBD	ELISA	CNI + MMF	univariate and multivariate regression analysis	
** **												mRNA-1273					
** **												mRNA-1273					
**Guarino (** 30)	2022	prospective	64.85 years (IQR, 57.2–70.09)	0.7541	14.08 years (IQR, 5.71–20.07)	HCC 192		336	444			BNT162b2	anti-Spike IgG	CLIA		multivariable analysis	Breakthrough
						ACLD 300											
						Viral infection 376											
						ALD 38											
						Autoimmune liver diseases 22											
						Other 50											
** **															CNI + MMF	univariate and multivariate regression analysis	
** **												mRNA-1273					
**Herrera (** 31)	2021	prospective	61.5 (18–88)	0.69	4.6 (0.3–26.8)		BMI 26.3	41	58			mRNA-1273	IgM/IgG antibodies		Monotherapy	logistic regression: vaccine unresponsiveness	Side effect
							HTN 33										
							DM 14										
							Leucopenia 12										
** **													ELISpot		Bitherapy		
** **															Triple therapy		
** **															Quadruple therapy		
**Huang** (32)	2022	NA	NA	NA	NA	NA	NA	44	86								
**Meunier** (33)	2022	retrospective	60 (SD: 13)	0.654	7.60±7.78	Alcohol 119 (36.4%)	DM 115			165	316	BNT162b2	Anti-spike antibody	Elecsys Anti-SARS-CoV-2 S (Roche) and SARS-CoV-2	Calcineurin inhibitor	univariate and multivariate analysis	serious adverse events: 0
						NASH 21 (6.4%)	BMI 26.3							IgG II Quant test (Abbott Laboratories)			
						HCC 11 (3.4%)											
						Auto-immune (PBC/AIH/PSC) 46 (14.1%)											
						Others 130 (39.8%											
** **												mRNA-1273			Mycophenolate mofetil use		
** **												AZD1222			Corticosteroid use		
**Nazaruk**	2021	retrospective					BMI 25					BNT162b2	anti-S1 Ab		mTOR inhibitor use		
** **																	
**Odriozola** (34)	2022	NA	60.6 (IQR, 56-28).	0.767	7.0 years (IQR, 4-12)			113	129	125	129	mRNA-1273	anti-SARSCoV-2 S1 antibodies				
**Marion** (35)	2021	NA	NA	NA	NA		Leu 5.6	31	65			BNT162b2 (99%)	Anti-SARSCov-2	ELISA		multivariate analysis	
													spike antibodies				
** **												mRNA-1273 (1%)					
**Rabinowich** (36)	2021	NA	60.1 ±12.8	0.7	76.6 ± 74.1	Viral 39	Leu count 6.23	38	80			BNT162b2	Anti-spike Ab	CLIA		Multivariable regression	side effects:1. systemic symptoms;2. Injection site reactions
						ALD 16	BMI 26.3										
						AIH 6	Htn 45										
						Hcc 3	DM 26										
						Others 8											
**Rahav** (37)	2021	prospective	68.0 (IQR: 51.0-71.0)	0.5277	7.0 [IQR 4.0-16.0]			25	36			BNT162b2	anti-RBD IgG	ELISA		Multivariable logistic regression	Side effect: local and systemic reactions
**Rashidi-Alavijeh** (38)	2021	cross-sectional study	57 (IQR: 49–64)	0.605	8 years (IQR 4–12)	HCC 10		34	43			BNT162b2	Anti-spike Ab	CLIA			
						ALD 7											
						Viral hep 3											
						Others 7											
**Saharia [34**	2022	prospective study					unspecified					8 weeks	neutralising antibodies				
**Sakai** (39)	2022	na	65	0.768				44	56			BNT162b2	anti-RBD IgG				
**Raszeja-Wyszomirska** (40)	2022	prospective study	54 [IQR: 19–74]	0.6406	NA		BMI 27	133	192			BNT162b2 (91%)	anti-spike IgG	CLIA		univariable analysis	
							DM 37										
**Ruether** (41)	2022	prospective study	55.0 ±13.19	0.572	median 7 years (IQR: 2-17)			102	138			mRNA-1273 (8%)	anti-S RBD IgG	ECLIA	CNI (92.8%); CNI monotherapy (23.9%);	logistic regression analysis	side effects
															prednisone (31.2%); CNI + prednisone (13.8%);		
															CNI + mTOR inhibitor (12.3%); CNI + MMF		
															(34.8%); CNI + azathioprine (6.5%); biologicals		
															(5.8%); ≥three agents (13%)		
** **												BNT162B2 (79.7%)	anti-S trimer				
** **												AZD1222 (12.3%)					
**Strauss** (42)	2021	na	Median: 64.0	0.429	Median: 6.9 years	NA	NA	130	161			BNT162b2 (53%)	anti-RBD antibody	ECLIA	antimetabolite		Breakthrough: 0%
			(IQR 48.0-69.0)		(IQR 2.9-15.0)												
** **												mRNA-1273 (47%)	anti-spike IgG				
**Tang** (43)	2022	retrospective study				Liver cancer 17											
						Wilson’s disease 1											
						Liver failure 12											
						Hepatic alveolar echinococcosis 2											
						Hepatitis cirrhosis 19											
						Drug-induced liver injury 3											
**Toniutto** (44)	2022	prospective study	67.3 (61.2-73.0)	0.72	91 months	HCC 41	DM 35 Htn 48 BMI 26	83	107	98	107	BNT162b2	anti-RBD	ECLIA	Tacrolimus (66.4%)	multivariate analysis	Systemic symptoms: 0 Breakthrough: 8.4%
															Cyclosporine (2.4%)		
															MMF (43.0%)		
															Everolimus (9.4%)		
															Prednisone (11.2%)		
** **																	pain at injection site: 11.2%
**Toniutto** (14)	2022	prospective study	57.9 (51.8-62.8)	0.702	94 (49–189) months			97	123			BNT162b2	anti-spike RBD	ECLIA	Tacrolimus (64.9%)	multivariate analysis	
** **															Cyclosporine (23.3%)		
** **															MMF (44.3%)		
** **															Everolimus (9.2%)		
** **															Prednisone (9.9%)		
**Tu** (45)	2022	prospective study	47 (40–53)	0.8571	2.4 (2.0–5.5)		BMI 23.8	6	35			inactivated vaccine	NA	NA	Tacrolimus (80%)		severe adverse events: 0

**Table 2:** Parameters included in the analyses.

Study	year	type	age	male	Time after LT, years	Etiology of LT	predominant comorbidities and presence of leukopenia or not, BMI	Comorbidities classification	BMI		factor predictor of poor serology (multivariate analysis)
SEBASTIAN	2022	Retrospective cohort	55 (46–63)	0.56	5.1 (1.8–9.7)		CVD 145 (36.2%)	Cardiovascular	low to normal		
							RESP 42 (10.5%)				NA
							DM 89 (22.2%)				
Pierluigi	2022	retrospective	57.9 (51.8-62.8)	92 (70.2)	94 (49-189)month	HCV 28	DM 46	Cardiovascular	high BMI	no leukopenia	age 60.5 (56.9-65.7)
						HBV 21	HTN 58				leukopenia 44
						NASH 0	Dyslipi 29				MMF 44
						AH 58	BMI 26				>2 IS drugs 44
						AI 13	Leu 5.649				univariate analysis
						Others 11	Neu 3.357				
							Asc 4				
Micaela	2022		58 (47–66)	357 (55.3%)	4.8 (1.3–9.5)			NS	NS		
Chombchanat	2022	prospective	14.5 ± 1.8	8 (66)	102 (58–160) months	Biliary atresia 7 (58.4)	BMI, kg/m2 (mean ± SD) 18.5 ± 2.8	NS	low to normal	no leukopenia	NA
						Autoimmune liver disease 1 (8.3)					
						Alagille syndrome 1 (8.3)					
						Others 3 (25)					

Ericka	2022	Prospective	58 [49–67]	40 (30.5%)	‘A		Coronary artery disease 7 (5.3%)	Cardiovascular	NS	NS	MMF OR= 14.0 [3.6–54.9]
											multivariate analysis

Anna	2002	retrospective	56 (42–65)	46 (46.9)	7 (3–13.3)	Alcoholic liver disease 14 (14.3) Autoimmune 25 (25.5)	24 (21.5–26.9)	Cardiovascular	low to normal	no leukopenia	Female sex
						Viral 15 (15.3	Diabetes 32 (32.7)				univariate analysis
						Hepatocellular carcinoma 8 (8.2)	Arterial Hypertension 52 Leucocytes 5.6 (4.1–7.3)				
Chauhan	2022							NS	NS	NS	
Chang	2022		60 IQR: (46–67)	0.56	6 IQR:			NS	NS	NS	NA
					(3–16)						
Balsby	2022	Prospective Cohort				NA	NA		NS		NA
Cholankeril	2021	prospective observational study	IQR: 63 (51-68)	0.7	3.3 (1.7-8.3)	ALD 24 (35)	Obesity* 37 (54)	Renal	high BMI	positive leukopenia	>2IS OR=3.10 (1.30-12.50)
						NASH 13 (19)	DM 33 (4				Age OR=1.04 [0.98-1.10]
						HCC 21 (30)	Chronic kidney disease stage III/IV‡ 36 (52)				time from transplant OR=1.02 [0.94-1.11]
							Leucopenia§ 9 (13				
John	2022		IQR: 68.9 (7.9)	0.976	IQR: 5.6 (5.0)	NA	NA	NS	NS	NS	NA
Cuadrado	2022	retrospective cohort	63 (IQR, 56–68)	0.767	7.0 years (IQR, 4–12)	Alcohol 56 (43.4)	Hypertension 78 (60.5)	Cardiovascular	low to normal	no leukopenia	>2 IS OR=0.07 (0.02–0.25)
						HCV 24 (18.6)	Diabetes 47 (36.4)				MMF OR=1.0 (1.0–1.0)
						Other 37 (28.7)	Chronic kidney disease 46 (35.9)				Leukopenia OR=1.0 (1.0–1.0)
						Hepatocellular carcinoma 52 (40.3	Chronic lung disease 12 (9.3)				
D’Offizi	2021	NA	59 (IQR 56-61)		6 years (IQR 3-10,			NS	NS	NS	MMF OR=1.60 (1.16-2.20) Time from transpl OR=2.19 (1.15-4.16) >2IS OR=1.6 [1.16-2.2]
					range 1-26)						
Davidov	2022	prospective	65 (IQR 52-70)	0.574	7 years (IQR 4-18)		DM 24	Renal	low to normal	no leukopenia	renal disease OR=7.1 [1.3-37.4]
							HTN 28				>2 IS drugs OR=10[2.5-50]
							CKD 39				low Egfr OR=7.10 [1.3-37.4]
							mBMI=25				
							Leu 5.6				
Davidov	2022	prospective	59 ± 15 (Mean+SD)	0.566	7 years (IQR,4-16)	NASH 13	Diabetes mellitus 31	Cardiovascular	low to normal	no leukopenia	
						Viral hepatitis 27	Hypertension 36				
						Others 23	Chronic kidney disease 25				
							BMI 25				
							Leu K/μL 6.0				
Fernández-Ruiz	2021		NA	NA	NA		Htn 32	Cardiovascular	high BMI	NS	univariate analysis
							DM 11				
							COPD 4				
							Obesity 22				
Furian	2022	prospective	64.0 ± 7.7	0.74	141.4 ± 204.9 months		NS	NS	NS	NS	NA
Giannella	2022	prospective	NA	NA	NA		NS	NS	NS	NS	Age OR=0.67 [0.6-0.76] Time to transplant 4.92 (2.56 9.45 >2 IS drugs 0.29 (0.20 0.43)
Harberts	2022	prospective	59.0 (51.0–68.3)	0.604	NA			NS	NS		lower eGFR OR=4.72 [1.56-14.38]
Guarino	2022	prospective	64.85 years (IQR, 57.2–70.09)	0.7541	14.08 years (IQR, 5.71–20.07)	HCC 192		Cardiovascular	NS	NS	Age OR=9.09 (1.16-0.76)
						ACLD 300					longer time to transplant OR=4.55 (1.24–16.60)
						Viral infection 376					MMF OR=0.51 (0.28–0.93)
						ALD 38					>2IS OR=0.58 [0.31-1.03]
						Autoimmune liver diseases 22					
						Other 50					
									NS	NS	
Herrera	2021	prospective	61.5 (18–88)	0.69	4.6 (0.3–26.8)		BMI 26.3	Cardiovascular	high BMI	positive leukopenia	leukopenia OR=5.5 (1.7–17.7)
							HTN 33				MMF OR=10.10 [2.3-44.3]
							DM 14				
							Leucopenia 12				
Huang	2022	NA	NA	NA	NA	NA	NA	NS	NS	NS	NA
Meunier	2022	retrospective	60 (SD: 13)	0.654	7.60±7.78	Alcohol 119 (36.4%)	DM 115	Endocrine	high BMI	NS	Male sex OR=2.247 [1.194 4.227]
						NASH 21 (6.4%)	BMI 26.3				MMF OR=2.18 [1.23 3.87]
						HCC 11 (3.4%)					multivariate analysis
						Auto-immune (PBC/AIH/PSC) 46 (14.1%)					
						Others 130 (39.8%					
Nazaruk	2021	retrospective					BMI 25	NS	high BMI	NS	NA
Odriozola	2022	NA	60.6 (IQR, 56-28).	0.767	7.0 years (IQR, 4-12)			NS	NS	NS	
Marion	2021	NA	NA	NA	NA		Leu 5.6	NS	NS	NS	male gender OR=1.964 [1.145-3.371]
											Tiime from trans OR=1.004 [1.001-1.007]
											eGFR1. OR=024 [1.011-1.037 >2 IS drugs OR= 2.463 [1.139-5.328] multivariate analysis
Rabinowich	2021	NA	60.1 ±12.8	0.7	76.6 ± 74.1	Viral 39	Leu count 6.23	Cardiovascular	high BMI	NS	>2 IS (OR 1.73; 95% CI 1.21–2.52)
						ALD 16	BMI 26.3				lower eGFR (OR 0.8; 95% CI 0.47–0.95)
						AIH 6	Htn 45				Age (OR 1.3; 95% CI 1.17–1.95;
						Hcc 3	DM 26				MMF OR=1.8 [1.15-3.47]
						Others 8					
Rahav	2021	prospective	68.0 (IQR: 51.0-71.0)	0.5277	7.0 [IQR 4.0-16.0]			NS	NS	NS	Age OR= 0.41 [0.30 0.57]
Rashidi-Alavijeh	2021	cross-sectional study	57 (IQR: 49–64)	0.605	8 years (IQR 4–12)	HCC 10		NS	NS	NS	NA
						ALD 7					
						Viral hep 3					
						Others 7					
Saharia	2022	prospective study					unspecified	NS	NS	NS	NA
Sakai	2022	na	65	0.768					NS		NA
Raszeja-Wyszomirska	2022	prospective study	54 [IQR: 19–74]	0.6406	NA		BMI 27	Endocrine	high BMI	NS	age OR=2.09 (1.04–4.20)
							DM 37				renal comorb OR=2.44 (1.27–4.67) MMF OR=2.99 (1.45–6.19) low eGFR OR=2.09 [1.04-4.19]
Ruether	2022	prospective study	55.0 ±13.19	0.572	median 7 years (IQR: 2-17)			NS	NS	NS	Age OR=4.57 [1.48-14.05] >2IS OR=1.78 [0.74-4.3]
Strauss	2021	na	Median: 64.0	0.429	Median: 6.9 years	NA	NA	NS	NS	NS	NA
			(IQR 48.0-69.0)		(IQR 2.9-15.0)						
Tang	2022	retrospective study				Liver cancer 17		NS	NS	NS	NA
						Wilson’s disease 1					
						Liver failure 12					
						Hepatic alveolar echinococcosis 2					
						Hepatitis cirrhosis 19					
						Drug-induced liver injury 3					
Toniutto	2022	prospective study	67.3 (61.2-73.0)	0.72	91 months		DM 35 Htn 48 BMI 26	Cardiovascular	high BMI	NS	MMF OR=0.211 [0.082-0.542 eGFR OR=1.078[ 1.020-1.139]
Toniutto	2022	prospective study	57.9 (51.8-62.8)	0.702	94 (49–189) months			NS	NS	NS	
Tu	2022	prospective study	47 (40–53)	0.8571	2.4 (2.0–5.5)		BMI 23.8	NS	NS	NS	

### 3.1 Overall seroconversion rates after SARS-CoV-2 vaccination in LT recipients

**Figure 2:**
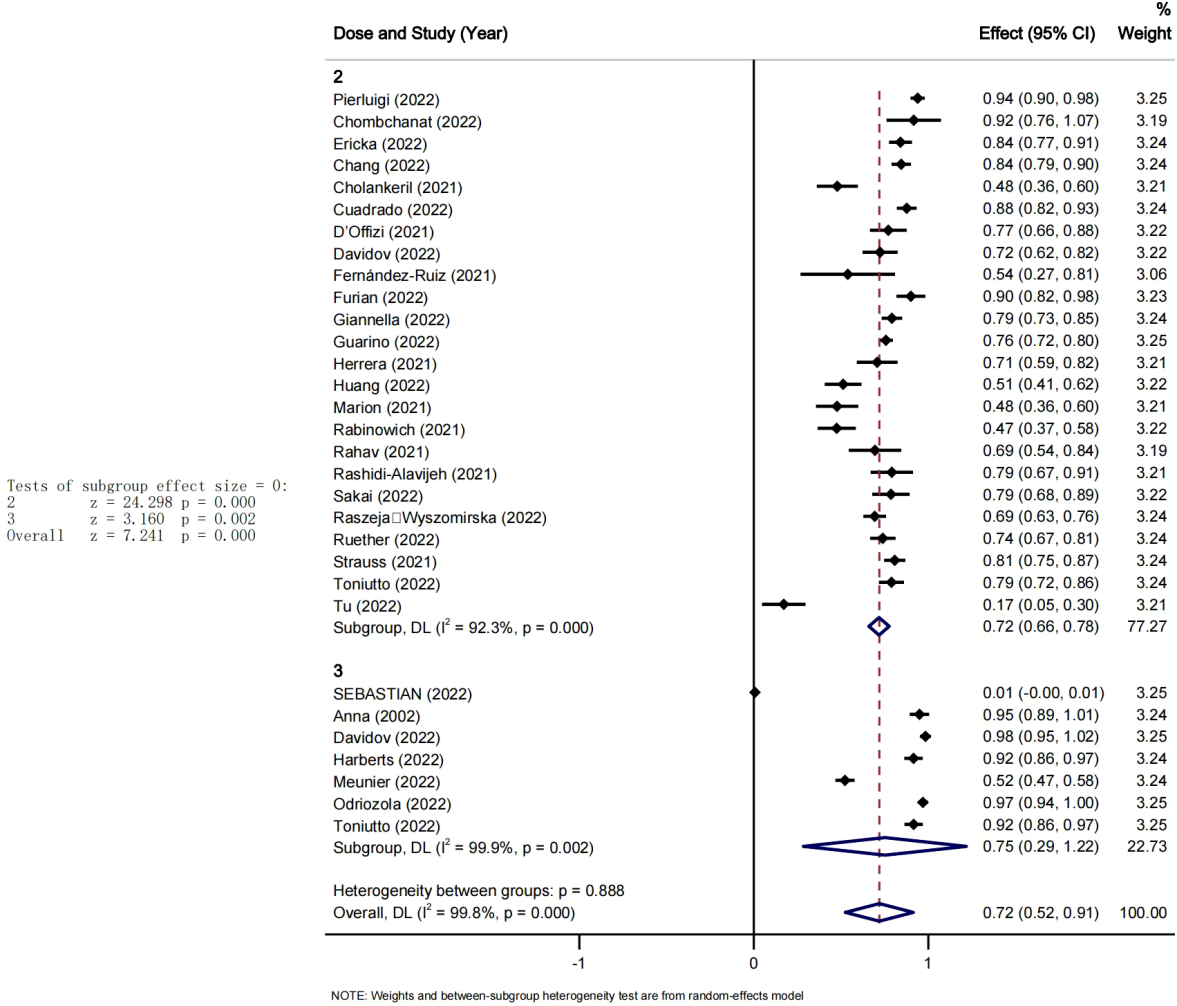
Seroconversion rates after second and third dose of SARS-CoV-2 vaccine LTR.

A summary of seroconversion rates after vaccination against SARS-CoV-2 is provided in **Figure 2**. All the included studies reported seroconversion following at least two doses of covid vaccine (13–20, 22–41, 45, 49, 50). The pooled seroconversion rate after at least two doses of SARS-CoV-2 vaccination was 72% (95% CI [0.52-0.91], P<0.05). With significant heterogeneity among studies I^2 = ^99.9%, and a non-significant heterogeneity between the two groups Pi=0.88. It could be observed that for studies reporting only two doses the seroconversion rate was about 72% (95%CI [0.66-0.75],P<0.05), whilst for studies reporting three doses of vaccine that rate was slightly higher around 75% (95%CI[0.29-1.22], P<0.05).

### 3.2 Subgroup analyses

We conducted subgroup analyses based on, major immunosuppressant drug used, BMI, major comorbidity, timing since transplantation and presence of leukopenia.

#### 3.2.1 BMI on seroconversion rate

Studies that included BMI reports in their participant baseline characteristics were grouped as lower to normal BMI and higher BMI categories; the rest of the studies that didn’t include BMI reports on their participants’ baselines were labeled as NS (not specified). As shown in **Figure S1**, the pooled seroconversion rate within the lower to normal BMI group was 74% (95% CI [0.22-1.27], P<0.05) against 67% (95% CI [0.52-0.81], P<0.05) in the high BMI group. Which could mean that LTR with normal BMI may present with a better seroconversion rate after at least two doses of covid vaccine than those with high BMI. Though the heterogeneity across studies was high i^2 ^= 99 but the heterogeneity between the subgroup was not significant Pi=0.632

#### 3.2.2 Major comorbidities on seroconversion rate

To explore whether the presence of some sort of comorbidities at the time of vaccination could affect the seroconversion rate, we spotted the most common comorbidities across included studies and we categorized studies based on the major comorbidity (ie the comorbidity presents in a great majority of the participants included in that study). In 11 studies the majority of participants had cardiovascular comorbidity (see **Table 1**), the predominance of renal comorbidity was found only in two studies and two studies included endocrine disease as major comorbidity; the rest of the studies didn’t have a report on comorbidities and were categorized as NS (not specified) (**Figure S2**). The pooled seroconversion rate was 70% for cardiovascular, 73% for renal, and 60% for endocrine. This suggests that the presence of endocrine comorbidities could affect the seroconversion rate in LTR after at least two doses of vaccine. Statics results were significant P<0.005 for endocrine and renal with high heterogeneity across studies and a no significant heterogeneity between the group groups Pi=0.52.

#### 3.2.3 Presence of leukopenia on seroconversion rate

Studies were further categorized as positive leukopenia, no leukopenia and NS (not specified) groups. As expected the pooled seroconversion rate in the “positive leukopenia’’ group was the slowest 59% 95%CI [0.37, 0.82] and the pooled seroconversion rate in the “no leukopenia’’ group was the highest and could reach 80% 95%CI [0.70, 0.90]; indicating that baseline leukopenia could influence the seroconversion rate in LTR after at least two doses of covid vaccine. All these results were significant P<0.005 with high heterogeneity across studies and a not significant heterogeneity between groups Pi=0.22 (see **Figure S3**).

#### 3.2.4 Use of immunosuppressive agents

**Figure 3:**
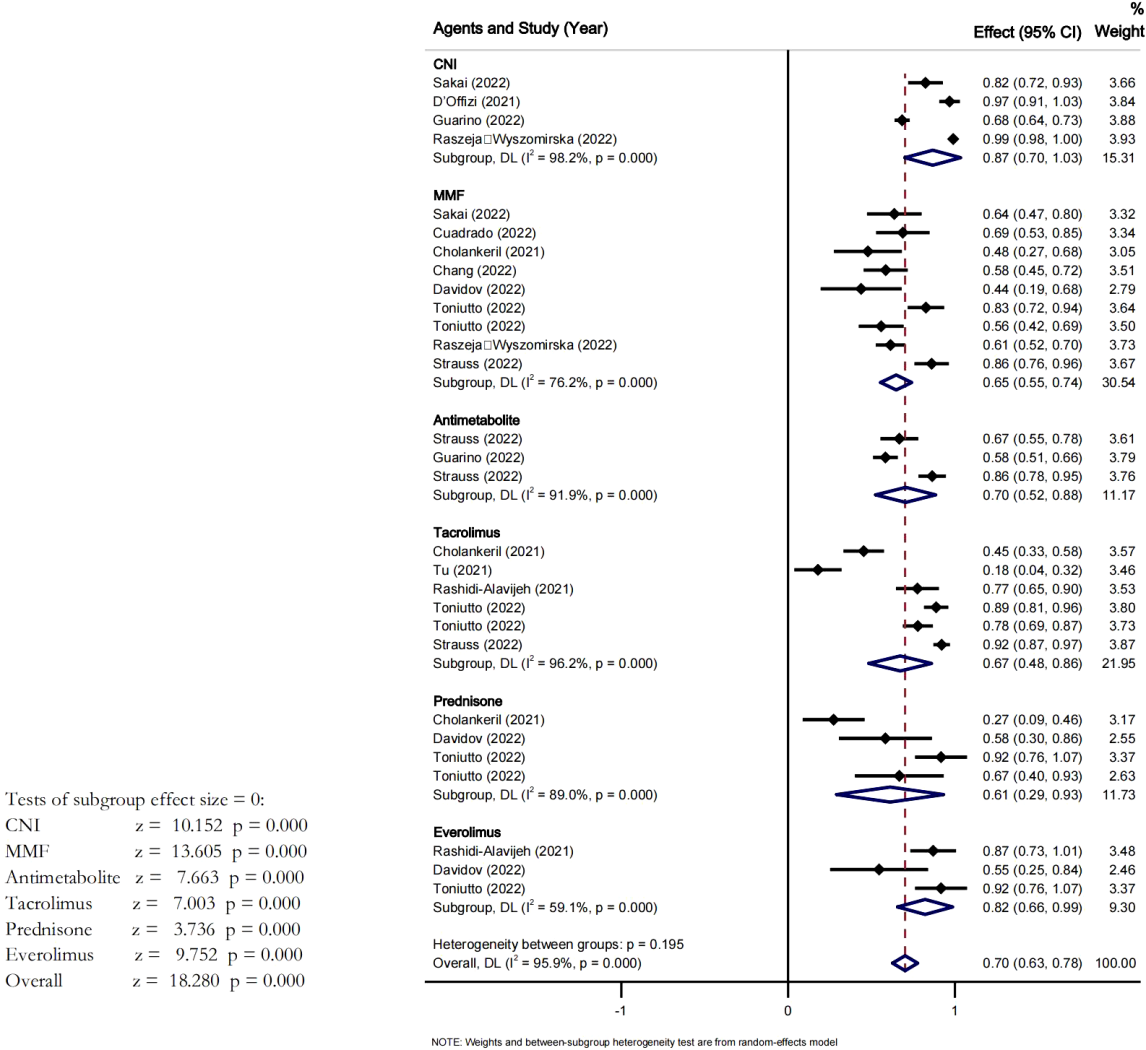
Effect of different drugs on the seroconversion rate of LTR after at least two dose of vaccine.

We conducted a subgroup analysis based on the use of immunosuppressive agents (**Figure 3**). After at least 2 doses of vaccines, the overall seroconversion rate in LT recipients treated with MMF was 65% (95% CI [0.55-0.74], I^2^ = 76%, P<0.05). The pooled seroconversion rate in patients on tacrolimus was 67% (95% CI 0.48-0.86], I^2^ = 96% P<0.05). The pooled seroconversion rate in patients on corticosteroids was 55% (95% CI [0.33-0.78], I^2^ = 77.0%). With everolimus the pooled seroconversion rate was 70% (95% CI [0.63-0.78], I^2^ = 59%, P<0.05). The seroconversion rates were 87% (95% CI [0.70-1.03], I^2^ = 98% P<0.05) and 70% (95% CI [0.52-0.88], I^2^ = 91%, P<0.05) for LT recipients treated with CNI and antimetabolite, respectively. There was a high heterogeneity across studies, however the heterogeneity among the subgroups was not significant Pi=0.195.

#### 3.2.5 Time since transplantation

**Figure 4:**
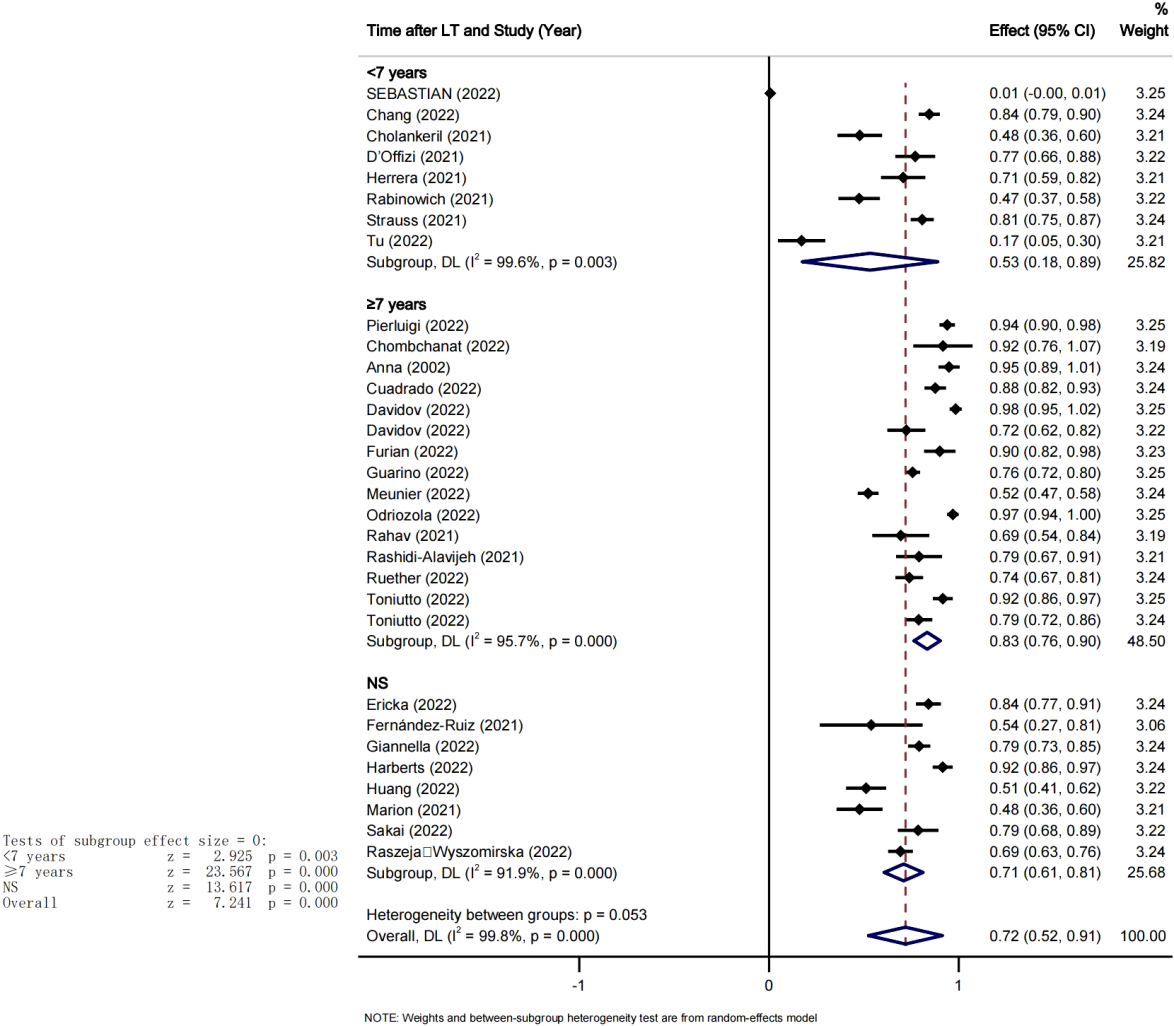
Vaccine seroconversion rates in LTR at different transplantation times.

We also conducted a subgroup analysis based on the mean time from transplant to vaccination. We computed the mean duration of time since transplantation across the included studies to use it as the cut-off point. Therefore, 7 years was set as the cut-off point and the studies were divided into two groups. The pooled seroconversion rate after at least 2 doses of COVID-19 vaccination was 53% (95% CI [0.18-0.83], P<0.05, I^2^ = 99.6%) in <7years group and 83% (95% CI [0.76-0.90], P<0.05 I^2^ = 95.7%) in > 7years group, the heterogeneity between the groups was significant Pi=0.005 (see **Figure 4**).

### 3.3 Predictors of poor serological response

**Figure 5:**
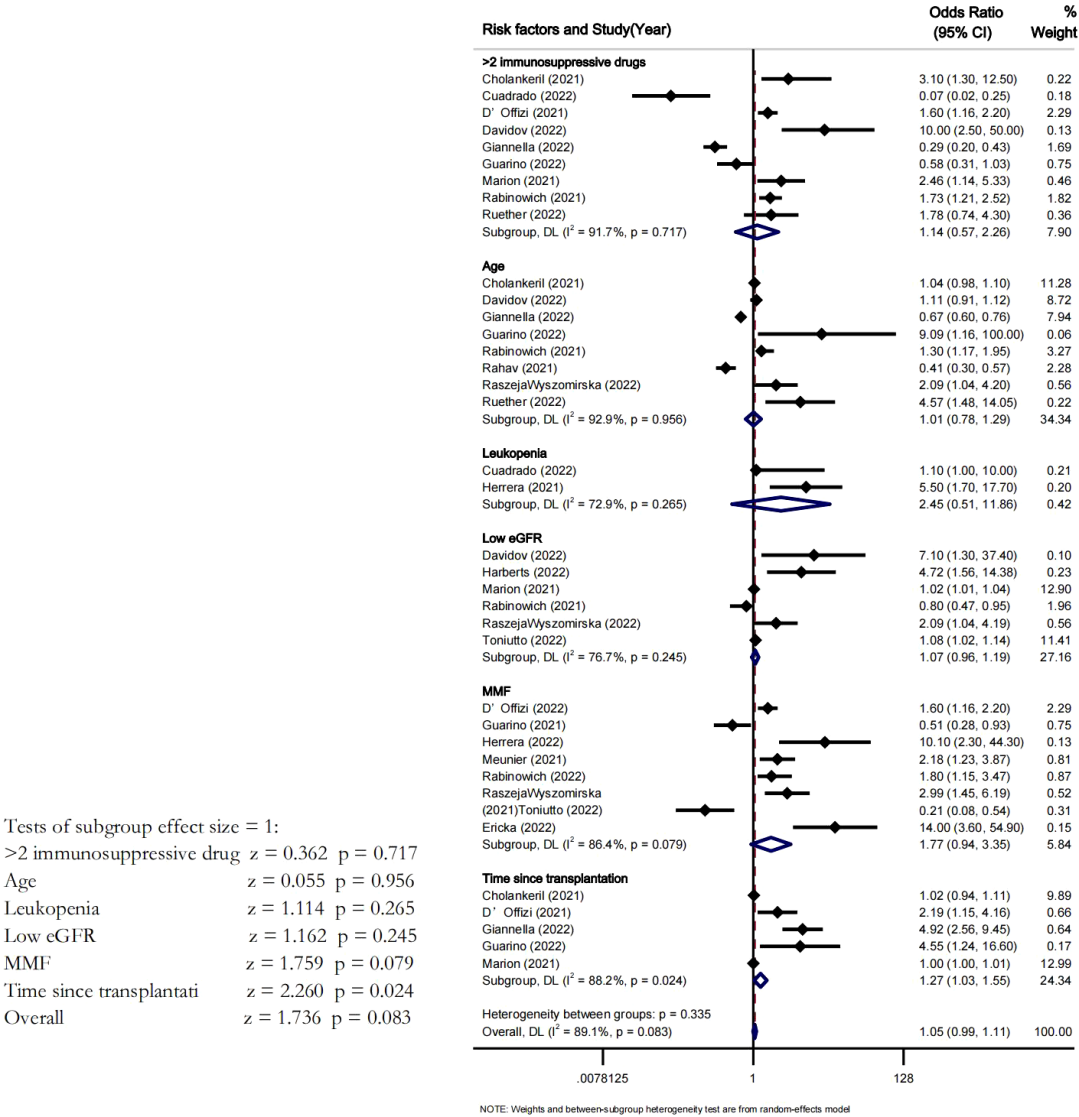
Risk factors predictor of poor immune Response.

Analysis was performed with some studies that had used multivariate analysis to identify predictors of poor serological response to COVID-19 vaccines. A total of 8 studies ^14,18,23,29,32,and 33^ included age as the potential risk factor in their multivariate analysis. Meta-analysis of these studies did confirm that advanced age was a risk factor for a poor serological response, but the association did not reach statistical significance (OR=1.01 95%CI [0.78-1.29] P=0.9). There pooled OR from the 8 studies that reported MMF as significant was also not statistically significant (P=0.07); 9 studies reported on the use of >2 immunosuppressive drugs, reported on decreased GFR, 5 on time since transplantation and 2 on leukopenia as the risk factors for poor immune response in multivariate analysis. However, our analysis results showed that only time since transplantation had reached statistical significance to be considered as risk factor for poor serological response (OR=1.27 95%CI [1.03-1.55], P=0.024) (**Figure 5**).

### 3.4 Breakthrough infections and adverse events

**Figure 6:**
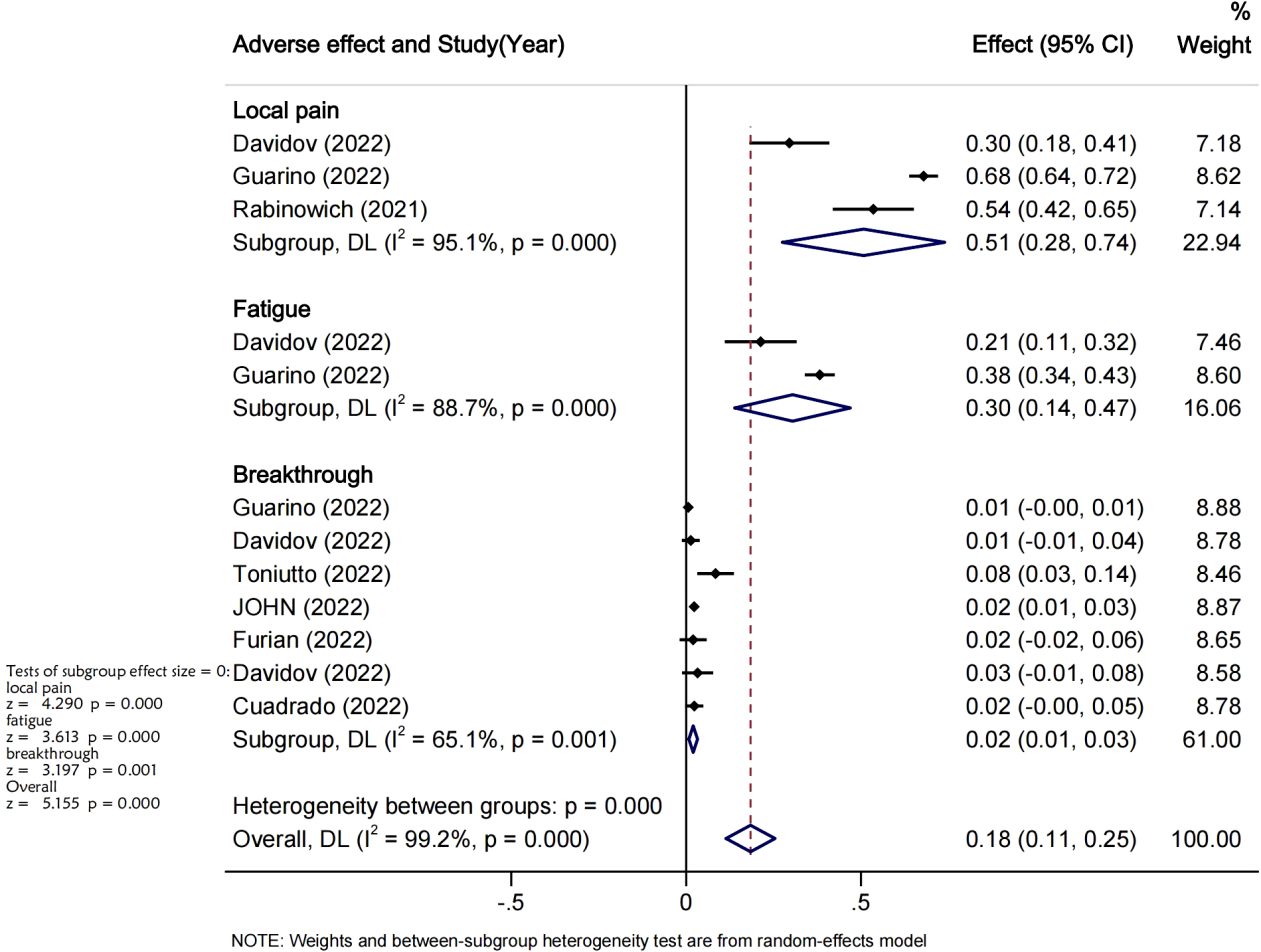
Adverse events and breakthrough infections analysis. Publication bias Moose checklist.

Seven studies reported on breakthrough infections after COVID-19 vaccination in LT recipients. In total, 44 infections and 2 deaths occurred in 2083 LT recipients during the follow-up period. The overall rate of breakthrough infections after complete vaccination was 2% (95% CI 0.01-0.03, P<0.05, I^2^ = 63.0%) (**Figure 6**). 3 studies reported adverse events after COVID-19 vaccination in LT recipients. The incidence of combined adverse events and breakthrough infections after COVID-19 vaccination was 18% (95%CI [0.11-0.25], I^2^ = 98.6%, P<0.05). The major adverse events recorded were local pain at the injection site with an incidence around 51% (95%CI [0.28-0.74], I^2^ = 97.9%, P<0.05) and fatigue which incidence was of 30%(95%CI: -[0.14%-0.47], I^2^ = 95%, P<0.05), Pi=0.00 (**Figure 6**).

### 3.5 Sensitivity analyses results

The pooled seroconversion rate and its 95% CI didn’t varie much when selected studies were omitted by the stata analysis software (72%,95%CI [0.52-0.90]. Which means that our results are reliable and were not just subject to chance. The details of all the sensitivity results are available attached to its figure generated in this analysis.

### 3.6 Publication bias

There was marked funnel plot asymetry confirmed by a positive Egger’s test P=0.000. This indicated that our results might be significantly impacted by threxisting publication bias among the included studies. See **Supplementary Files**.

## 4 Discussion

COVID-19 is now considered a global pandemic, currently causing millions of deaths in just a few years. At present, the pathogenesis and treatment of this disease are still under study. The widespread use of vaccines is currently considered the most important measure to control the pandemic (51).

Sars-CoV-2 infection results in an altered host immune response. T and N K cell depletion may occur during SARS-COV-2 infection (52). For transplant patients, long-term use of immunosuppressive agents may lead to an increased risk of SARS-CoV-2 infection. Since immunocompromised people are at high risk of severe disease and death after infection with a Novel coronavirus, it is recommended that solid organ transplant patients (hereinafter referred to as SOT recipients) receive the SARS-CoV-2 vaccine. In addition, many clinical studies on vaccines often exclude organ transplant patients, including a large proportion of liver transplant patients. Hence, the efficacy and safety of vaccination in liver transplant patients deserve further evaluation.

Among liver-transplant recipients, the pooled seroconversion rates was 72% (95% CI 0.66-0.78) after two doses of the SARS-CoV-2 vaccine, and there was a slight positive increase in the pooled seroconversion rate after three doses of vaccine (75% 95% CI: [0.73-1.00]), which was statistically significant. These results not only suggest that a third dose could be recommended for patients undergoing liver transplantation, even if the second dose improve the rate of seroconversion but also indicate that the immune response to the SARS-CoV-2 vaccine is not attenuated after the administration of a multiple-dose regimen in liver transplant recipients, despite prolonged use of immunosuppressive agents.

Though Previous meta-analysis did evaluate the seroconversion rate after two doses of vaccine in LTR, and evaluated on factors such as types of vaccine, the number of doses, time between booster doses, time since transplantation and presence of adverse events and breakthrough infections (53, 54). However the role of BMI and the presence or types of comorbidities on the seroconversion rate in LTR have not well been clarified in their pooled analysis and for the other factors their conclusion brought non-negligible feedback from the scientific community; therefore this matter may not yet as per say be considered as concluded and more investigation were necessary to validate previous theories made. In Our analysis the pooled seroconversion rate from studies characterized by a lower to normal BMI value was 74% (95% CI [0.22-1.27], P=0.005) against 67% (95% CI [0.52-0.81], P=0.000) in those characterized by high BMI value. This could imply that obese and overweight LTR may have a lower seroconversion rate after at least two doses of mRna vaccine. This may be explained by the fact that COVID-19 and obesity are intertwined pandemics. Those with obesity have a higher risk of severe outcomes from SARS-CoV-2 and excess weight increases the risk of adverse outcomes, regardless of comorbidities. Obesity affects metabolism causing insulin resistance, changes in adipokines (leptin increase, adiponectin decrease), and chronic inflammation. This leads to endothelial dysfunction and worsens a prothrombotic state. Obese mice shed viruses for longer, had more bacterial infections, and more respiratory damage. People with obesity may have reduced vaccine effectiveness due to the altered cytokine production and immune responses. This has been observed with influenza vaccine and preliminary reports also suggest that lower antibody concentrations can be found in obese patients after COVID-19 vaccine.

The comorbidities analysis revealed that the seroconversion rate after at least two doses of vaccine was lower in the group having endocrine pathologies as major comorbidity than the rest; however, this observation might actually not reflect the reality because of the few number of studies, howbeit previous records acknowledged that there was a risk of cardiac tissue damage following the SARS COV2 infection. Based on these facts it would be logical to assume that the presence of comorbities in LTR may affect the response to covid 19 vaccine.

In our subgroup analyses we also evaluated the influence of the presence of baseline leukopenia on the seroconversion rate and we found that as expected the pooled seroconversion rate in the “positive leukopenia’’ group was the lowest 59% and the pooled seroconversion rate in the “no leukopenia’’ group was the highest and could reach 80%. The goal of vaccination is generally to induce the formation of memory cells to prepare the body for future eventual invasion from the pathogen. Therefore, a good immunological status which could be marked by sufficient level of immunoglobulins and immune cells is essential for optimized response after vaccination. So in LTR with altered baseline immunity the positive response from the vaccine would appear to be lower than that of those with normal baseline immunity profile.

This analysis also showed that after at least two doses of vaccine in LTR the seroconversion rate was slightly higher in patients treated with CNI, and was low in patients treated with MMF, Tacrolimus and Prednisone. Therefore, CNI seemed to be the more appropriate immunosuppressant drugs for LTR after at least two vaccine doses. Studies have shown that for LTR, immunosuppressive treatment with different types of immunosuppressants (which possess different properties and mechanisms) is not a contraindication for vaccination. Therefore, vaccination remains feasible despite immunosuppressant therapy. Our findings also supported this theory. T cells are frequently inhibited by immunosuppressive drugs, and vaccine-induced immune responses also include T-cells. So the normal expectation would be a reduced seroconversion rate after vaccine.This shows that our analysis results might not be conclusive and more precise and well elaborated studies could re-evaluate, the characteristic of the immune response in patients taking immunosuppressant therapy.

. The study showed a remarkable difference in the concomitant seroconversion rate after two doses of the COVID-19 vaccine between patients with less than 7 years after liver transplantation and those with more than 7 years after liver transplantation. Studies have shown that the number of CD4~+CD25~+Foxp3~+ regulatory T cells in peripheral blood of patients who survived 6 months to 3 years, 3 years to 10 years, and more than 10 years after liver transplantation with immunosuppressive agents gradually decreased and maintained at a relatively low level with the extension of survival time (55). It is suggested that the immune response is different in patients with different survival times after transplantation. The time of transplantation also may have an impact on the vaccine conversion rate. The longer the time from transplantation, the better the vaccine seroconversion rate. It could also be said that the immune response in transplant patients may not be reduced with the extended survival time.

Another important measure of the effectiveness of SARS-CoV-2 vaccines is the incidence of breakthrough infections after vaccination against COVID-19. However, only 7 studies have evaluated infection following breakthrough infection S A R S-COV-2 infection; therefore, further research on the risk of breakthrough infection in liver transplant patients is warranted. Further studies are needed to assess the breakthrough infection rate after vaccination with SARS-COV-2 in LTR patients compared with the general population.

In addition, this meta-analysis also illustrated that the majority of adverse events after vaccine were limited to injection site side effects. Three studies reported that the major adverse events in LT recipients after COVID-19 vaccination were local pain at the injection site 51% (95%CI [0.28-0.74], I^2^ = 95%) and Systemic adverse events reported only in a small subset such as fatigue 30% (95%CI: [0.14-0.47], I^2^ = 88%). overall the incidence of adverse events following vaccination was very low and the majority did not require hospitalization. These results are suggesting that COVID-19 vaccination is safe for LTR patients.

The strength of our study is the meta-analysis of a large number of prospective studies that included data on COVID-19 vaccination in a large number of liver transplant recipients. In addition, subgroup analyses add to the robust statistical design. But there are limitations to this meta-analysis. Heterogeneity in immunosuppressive therapies, vaccine technics, regional differences in vaccination sites according to countries, may have also accounted for the heterogeneity across studies making it difficult to ascertain the assessment of some outcomes. The assessment of adverse events also presented some limitations, in part because of inherent limitations from the included studies used to assess adverse events and the small sample size, from the lack of randomized controls to accurately evaluate the incidence of adverse events after vaccination. The studies used different assays to assess SARS-CoV-2 antibodies, this may also have influenced the results. Therefore, further studies are needed to compare seroconversion rates between different assays for SARS-CoV-2 antibodies. Moreover, it is worth noting that infections could still occur despite the seroconversion post-vaccination 24, 39, 44 due to the different epidemic situations in different countries. Nevertheless, vaccination can reduce the severity of the disease. The T-cell response is also an important component of the SARS-COV-2 vaccine response (56). The first edition of the Technical Guidelines for SARS-CoV-2 Vaccination recommended inactivated and recombinant subunit vaccines for solid organ transplant patients (57). However, with the development of vaccines, mRNA vaccines have been widely used worldwide, and the difference in effectiveness among mRNA vaccines has attracted attention (8, 19, 27). For the immune response after 3 doses of the COVID-19 vaccine, the seroconversion rate of the mixed vaccine did not change much, while the seroconversion rate of the BNT162b2 and mrNA-1273 vaccines increased significantly. It is recommended to avoid mixed vaccine regimens whenever possible _53_. In addition, others limitations may reside in the inaccessibility of records published in language other than English and unpublished studies. Nonetheless this study is to our knowledge the first meta-analysis on immunogenicity of mRNA covid-vaccine in LTR that included BMI, Leukopenia and associated comorbidities in its analysis. Thus, making it worth of consideration.

## 5 Conclusion

In conclusion, this meta-analysis showed that the overall seroconversion rate of liver transplant recipients vaccinated with COVID-19 increased after booster vaccination, irrespective of vaccine type, immunosuppressant used. This meta-analysis also demonstrated that normal BMI, absence of pre vaccination leukopenia and increased duration of transplantation was significantly positively also associated with improved seroconversion rate. Further studies are needed to investigate on the efficacy of different vaccines against SARS-CoV-2 variants infection in LTR and the T-cell response after covid19 vaccination

## Data availability statement

The original contributions presented in the study are included in the article/**Supplementary Material**. Further inquiries can be directed to the corresponding author.

## Author contributions

YX designed and supervised the study, ZY and XL collected data, XL and FL performed the analysis and drafted the manuscript, and FL proofread the paper. All authors contributed to the article and approved the submitted version.

## Supplementary material

Supplementary Figure 1 Effect of BMI on the seroconversion rate of LTR second dose vaccine.

Supplementary Figure 2 Effect of comorbidities on the seroconversion rate of LTR after at least two doses of vaccine.

Supplementary Figure 3 Presence of leukopenia on seroconversion rate.
