# Supplementary material for: Factors influencing immunogenicity and safety of SARS-CoV-2 vaccine in liver transplantation recipients: a systematic review and meta-analysis
Source: Front Immunol. 2023 Sep 5;14:1145081. doi: 10.3389/fimmu.2023.1145081 (PMC10508849; doi:10.3389/fimmu.2023.1145081)
Supplement: Supplementary Figure 1 — Effect of BMI on the seroconversion rate of LTR second dose vaccine. [file Image_1.pdf]

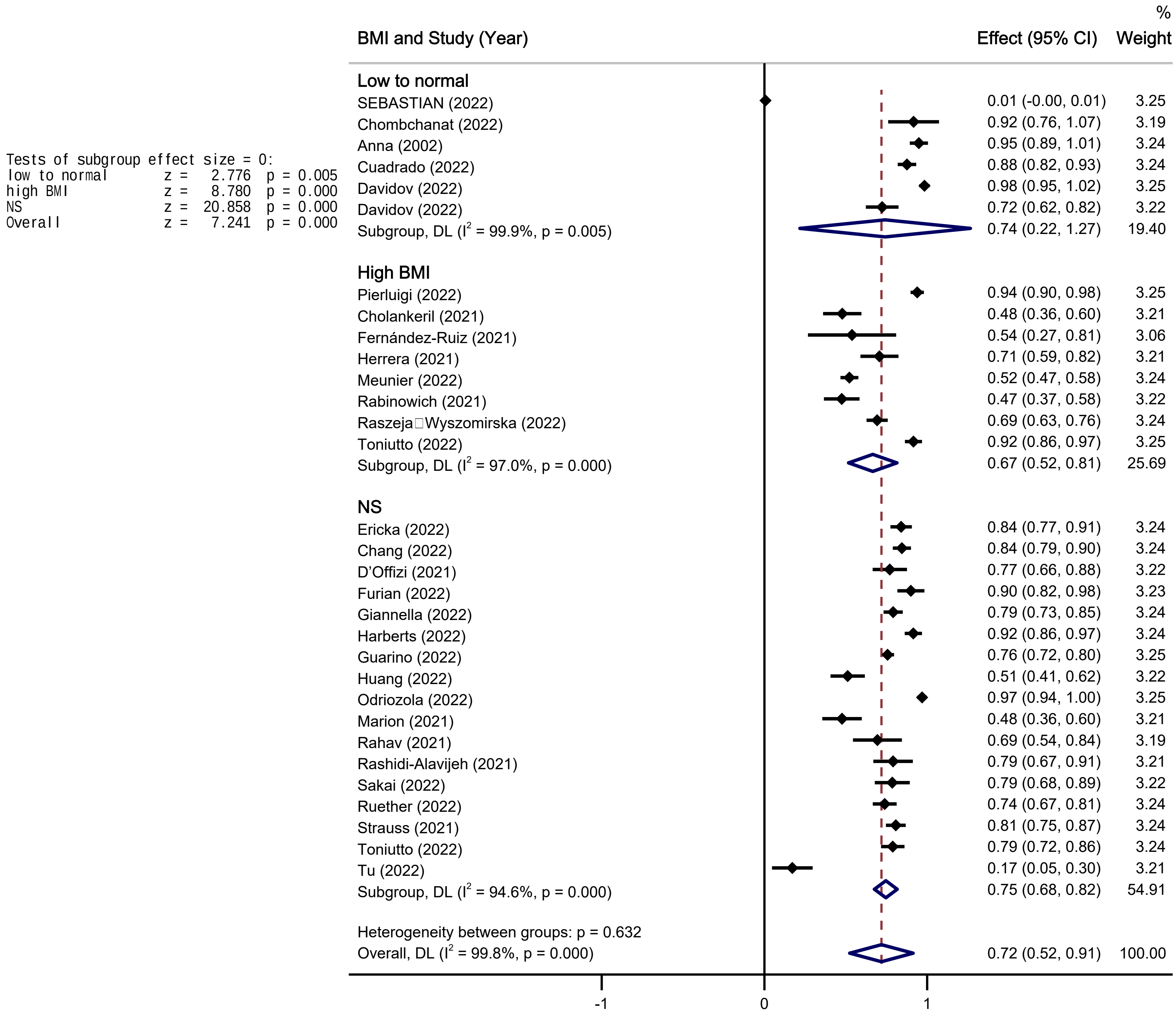

NOTE: Weights and between-subgroup heterogeneity test are from random-effects model

Study omitted	Estimate	[95% Conf.	Interval]
SEBASTIAN (2022)	.89658433	.81825805	.97491062
Chombchanat (2022)	.70759684	.13345277	1.2817409
Anna (2002)	.70055038	.12409452	1.2770063
Cuadrado (2022)	.71525139	.12966834	1.3008344
Davidov (2022)	.69346803	.14751282	1.2394233
Davidov (2022)	.74576265	.16394611	1.3275791
Nazaruk (2021)	.74207491	.21820916	1.2659407
Combined	.74207493	.21820916	1.2659407

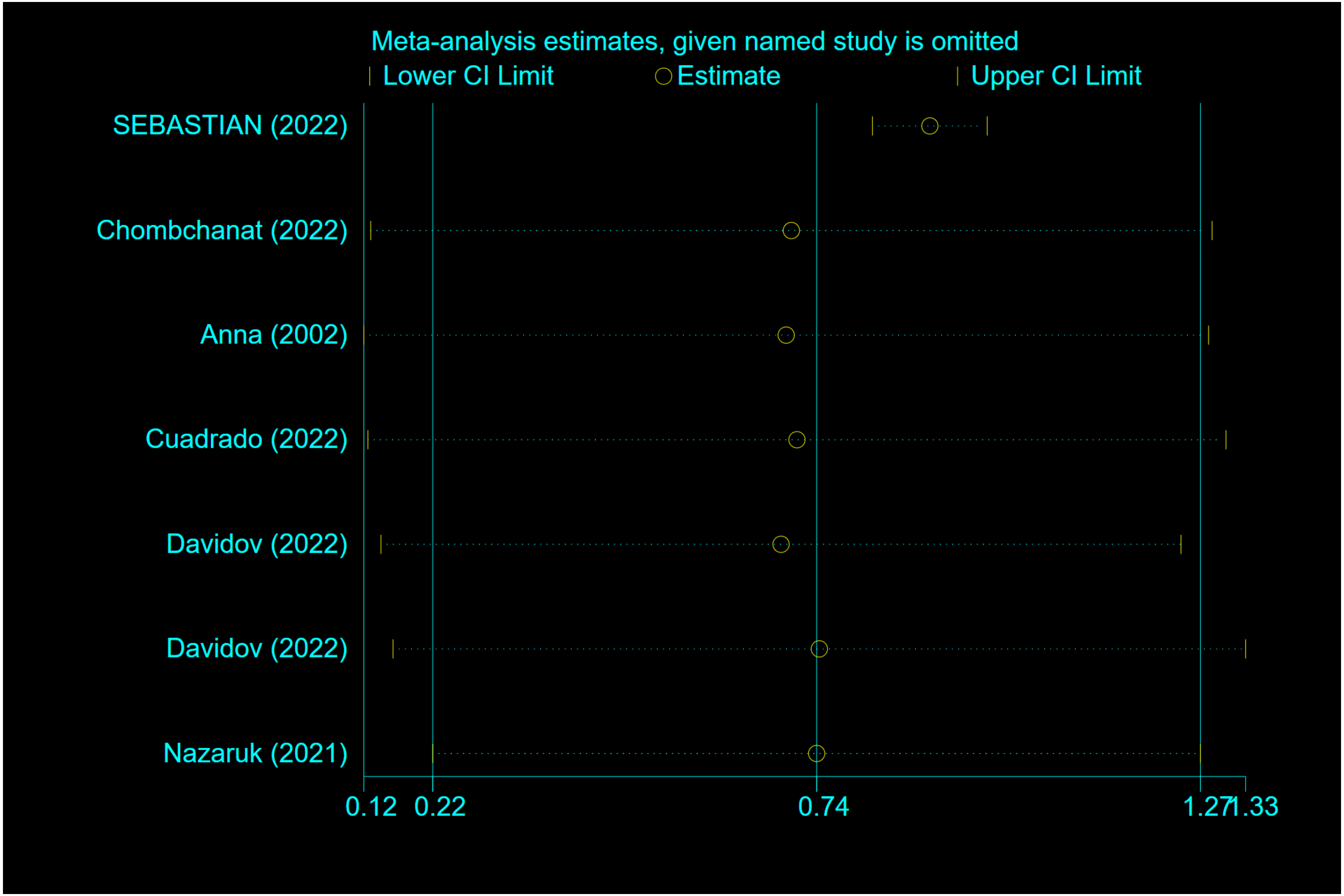
Meta-analysis estimates, given named study is omitted
Lower CI Limit
Estimate
Upper CI Limit
SEBASTIAN (2022)
Chombchanat (2022)
Anna (2002)
Cuadrado (2022)
Davidov (2022)
Davidov (2022)
Nazaruk (2021)
0.12
0.22
0.74
1.27
1.33

Study omitted	Estimate	[95% Conf.	Interval]
**Pierluigi (2022)**	**.62433398**	**.47408319**	**.77458477**
**Cholankeril (2021)**	**.6929509**	**.53748685**	**.8484149**
**Fernández-Ruiz (2021)**	**.67902541**	**.52263057**	**.83542019**
**Herrera (2021)**	**.65964663**	**.49597141**	**.82332182**
**Meunier (2022)**	**.69027489**	**.54860073**	**.83194906**
**Rabinowich (2021)**	**.69386756**	**.53926206**	**.84847307**
**Raszeja�Wyszomirska (2022)**	**.66069233**	**.48706216**	**.83432257**
**Toniutto (2022)**	**.62697679**	**.45721108**	**.79674244**
**Combined**	**.66592298**	**.51727662**	**.81456934**

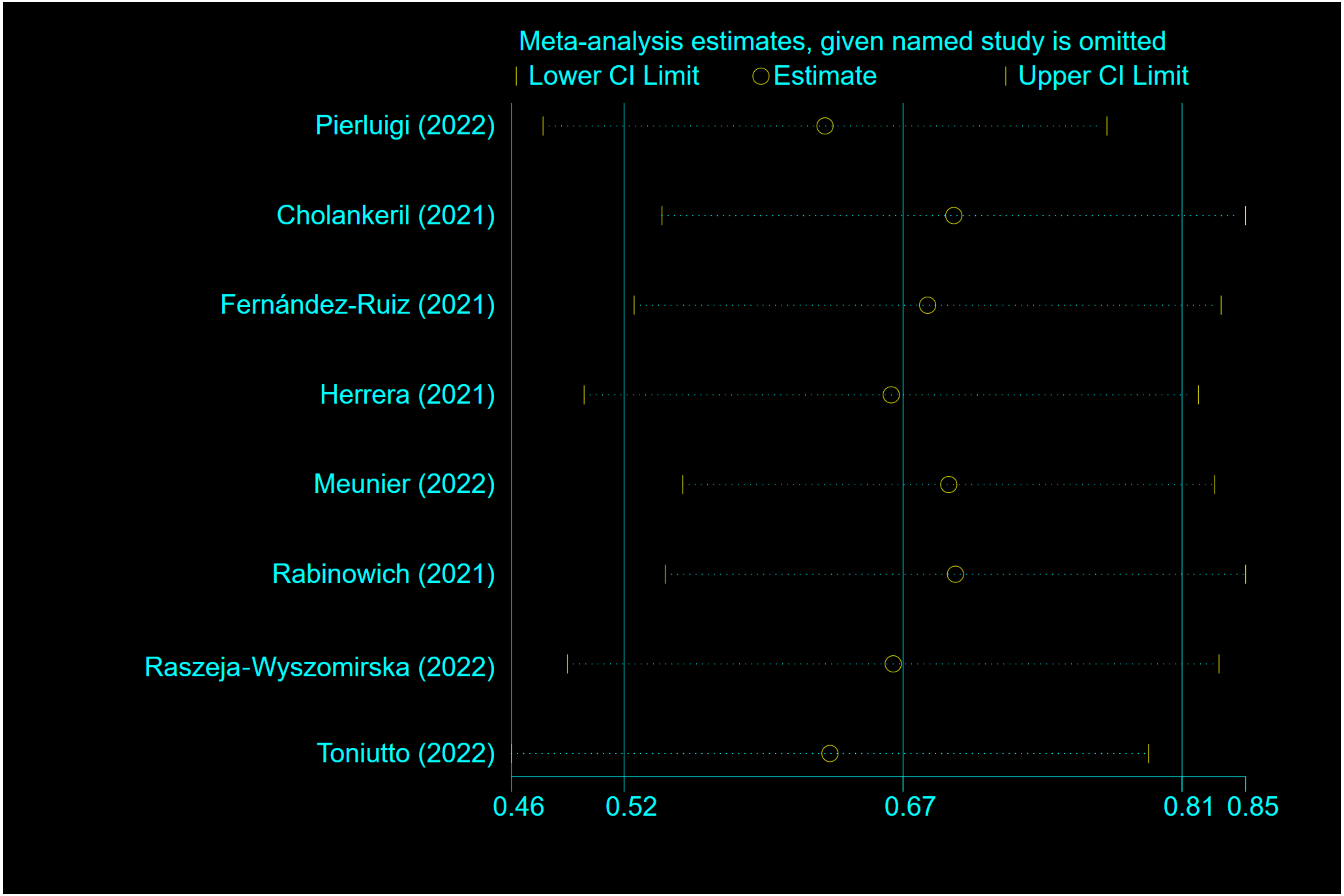

Meta-analysis estimates, given named study is omitted
Lower CI Limit
Estimate
Upper CI Limit
Pierluigi (2022)
Cholankeril (2021)
Fernández-Ruiz (2021)
Herrera (2021)
Meunier (2022)
Rabinowich (2021)
Raszeja-Wyszomirska (2022)
Toniutto (2022)
0.46
0.52
0.67
0.81
0.85
