# Supplementary material for: Factors influencing immunogenicity and safety of SARS-CoV-2 vaccine in liver transplantation recipients: a systematic review and meta-analysis
Source: Front Immunol. 2023 Sep 5;14:1145081. doi: 10.3389/fimmu.2023.1145081 (PMC10508849; doi:10.3389/fimmu.2023.1145081)
Supplement: Supplementary Figure 2 — Effect of comorbidities on the seroconversion rate of LTR after at least two doses of vaccine. [file Image_2.pdf]

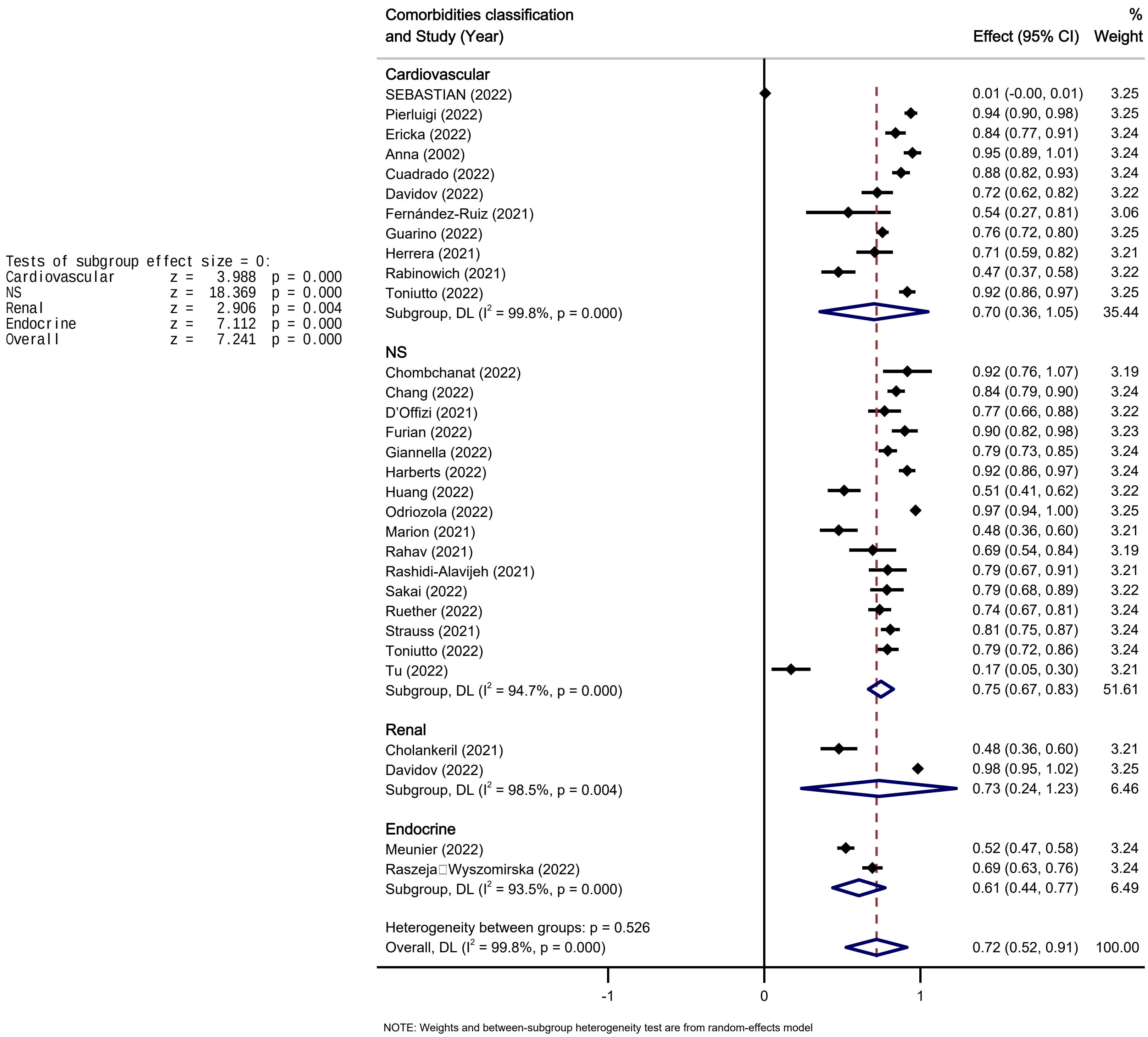
Tests of subgroup effect size = 0:
Cardiovascular z = 3.988 p = 0.000
NS z = 18.369 p = 0.000
Renal z = 2.906 p = 0.004
Endocrine z = 7.112 p = 0.000
Overall z = 7.241 p = 0.000
Comorbidities classification and Study (Year)
Effect (95% CI)
% Weight
Cardiovascular
SEBASTIAN (2022) 0.01 (-0.00, 0.01) 3.25
Pierluigi (2022) 0.94 (0.90, 0.98) 3.25
Ericka (2022) 0.84 (0.77, 0.91) 3.24
Anna (2002) 0.95 (0.89, 1.01) 3.24
Cuadrado (2022) 0.88 (0.82, 0.93) 3.24
Davidov (2022) 0.72 (0.62, 0.82) 3.22
Fernández-Ruiz (2021) 0.54 (0.27, 0.81) 3.06
Guarino (2022) 0.76 (0.72, 0.80) 3.25
Herrera (2021) 0.71 (0.59, 0.82) 3.21
Rabinowich (2021) 0.47 (0.37, 0.58) 3.22
Toniutto (2022) 0.92 (0.86, 0.97) 3.25
Subgroup, DL (I^2 = 99.8%, p = 0.000) 0.70 (0.36, 1.05) 35.44
NS
Chombchanat (2022) 0.92 (0.76, 1.07) 3.19
Chang (2022) 0.84 (0.79, 0.90) 3.24
D'Offizi (2021) 0.77 (0.66, 0.88) 3.22
Furian (2022) 0.90 (0.82, 0.98) 3.23
Giannella (2022) 0.79 (0.73, 0.85) 3.24
Harberts (2022) 0.92 (0.86, 0.97) 3.24
Huang (2022) 0.51 (0.41, 0.62) 3.22
Odriozola (2022) 0.97 (0.94, 1.00) 3.25
Marion (2021) 0.48 (0.36, 0.60) 3.21
Rahav (2021) 0.69 (0.54, 0.84) 3.19
Rashidi-Alavijeh (2021) 0.79 (0.67, 0.91) 3.21
Sakai (2022) 0.79 (0.68, 0.89) 3.22
Ruether (2022) 0.74 (0.67, 0.81) 3.24
Strauss (2021) 0.81 (0.75, 0.87) 3.24
Toniutto (2022) 0.79 (0.72, 0.86) 3.24
Tu (2022) 0.17 (0.05, 0.30) 3.21
Subgroup, DL (I^2 = 94.7%, p = 0.000) 0.75 (0.67, 0.83) 51.61
Renal
Cholankeril (2021) 0.48 (0.36, 0.60) 3.21
Davidov (2022) 0.98 (0.95, 1.02) 3.25
Subgroup, DL (I^2 = 98.5%, p = 0.004) 0.73 (0.24, 1.23) 6.46
Endocrine
Meunier (2022) 0.52 (0.47, 0.58) 3.24
Raszeja□Wyszomirska (2022) 0.69 (0.63, 0.76) 3.24
Subgroup, DL (I^2 = 93.5%, p = 0.000) 0.61 (0.44, 0.77) 6.49
Heterogeneity between groups: p = 0.526
Overall, DL (I^2 = 99.8%, p = 0.000) 0.72 (0.52, 0.91) 100.00
-1
0
1
NOTE: Weights and between-subgroup heterogeneity test are from random-effects model

Study omitted	Estimate	[95% Conf.	Interval]
SEBASTIAN (2022)	.79409754	.71846992	.86972523
Pierluigi (2022)	.67955691	.32500833	1.0341055
Ericka (2022)	.68949014	.32506424	1.053916
Anna (2002)	.67854309	.31997764	1.0371085
Cuadrado (2022)	.68589592	.32283297	1.0489588
Davidov (2022)	.70122862	.33619633	1.0662608
Fernández-Ruiz (2021)	.71894705	.35658196	1.0813121
Guarino (2022)	.69783354	.32081562	1.0748515
Herrera (2021)	.70291209	.33842483	1.0673994
Rabinowich (2021)	.72607607	.35928491	1.0928673
Toniutto (2022)	.68187916	.32158881	1.0421696
Combined	.70328154	.35760511	1.048958

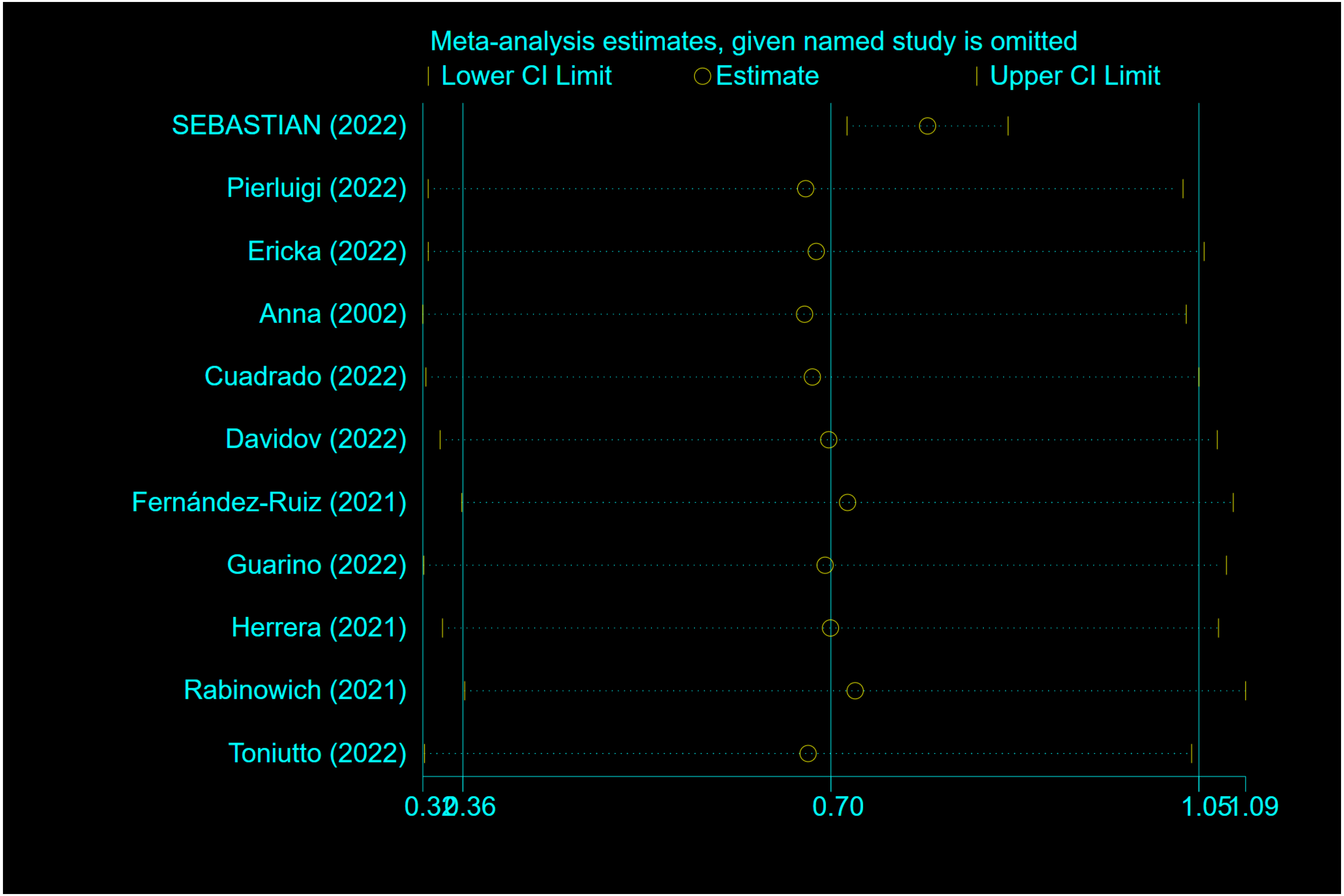
Meta-analysis estimates, given named study is omitted
Lower CI Limit
Estimate
Upper CI Limit
SEBASTIAN (2022)
Pierluigi (2022)
Ericka (2022)
Anna (2002)
Cuadrado (2022)
Davidov (2022)
Fernández-Ruiz (2021)
Guarino (2022)
Herrera (2021)
Rabinowich (2021)
Toniutto (2022)
0.32
0.36
0.70
1.05
1.09

Study omitted	Estimate	[95% Conf	Interval]
**Cholankeril (2021)**	**.98360658**	**.9517405**	**1.0154727**
**Davidov (2022)**	**.4782609**	**.3603965**	**.5961253**
**Combined**	**.73425028**	**.23906329**	**1.2294373**

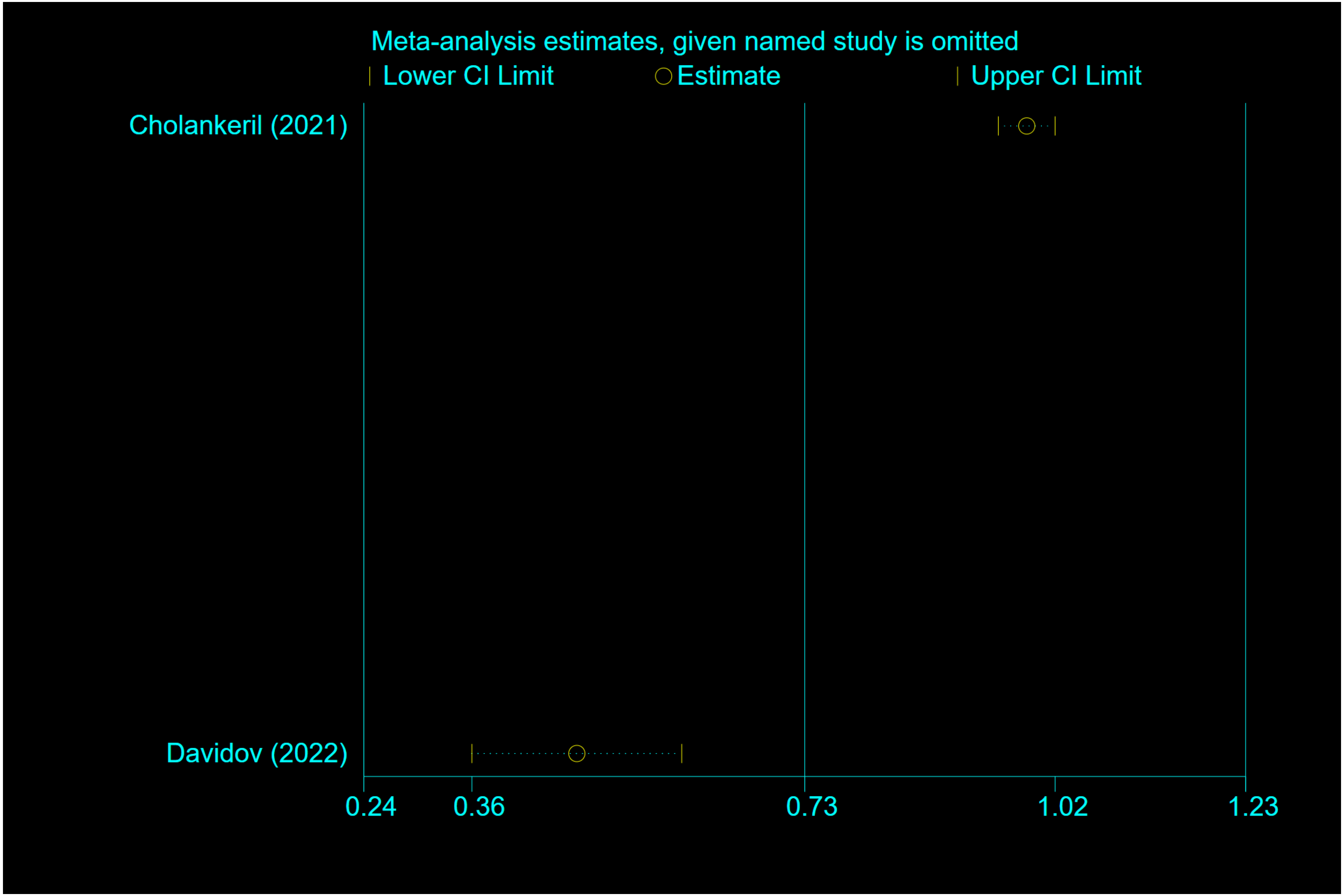
Meta-analysis estimates, given named study is omitted
Lower CI Limit
Estimate
Upper CI Limit
Cholankeril (2021)
Davidov (2022)
0.24
0.36
0.73
1.02
1.23

Study omitted	Estimate	[95% Conf.	Interval]
Meunier (2022)	.69270831	.6274482	.75796843
Raszeja□Wyszomirska (2022)	.52215189	.46707767	.5772261
Combined	.6064947	.43936253	.77362687

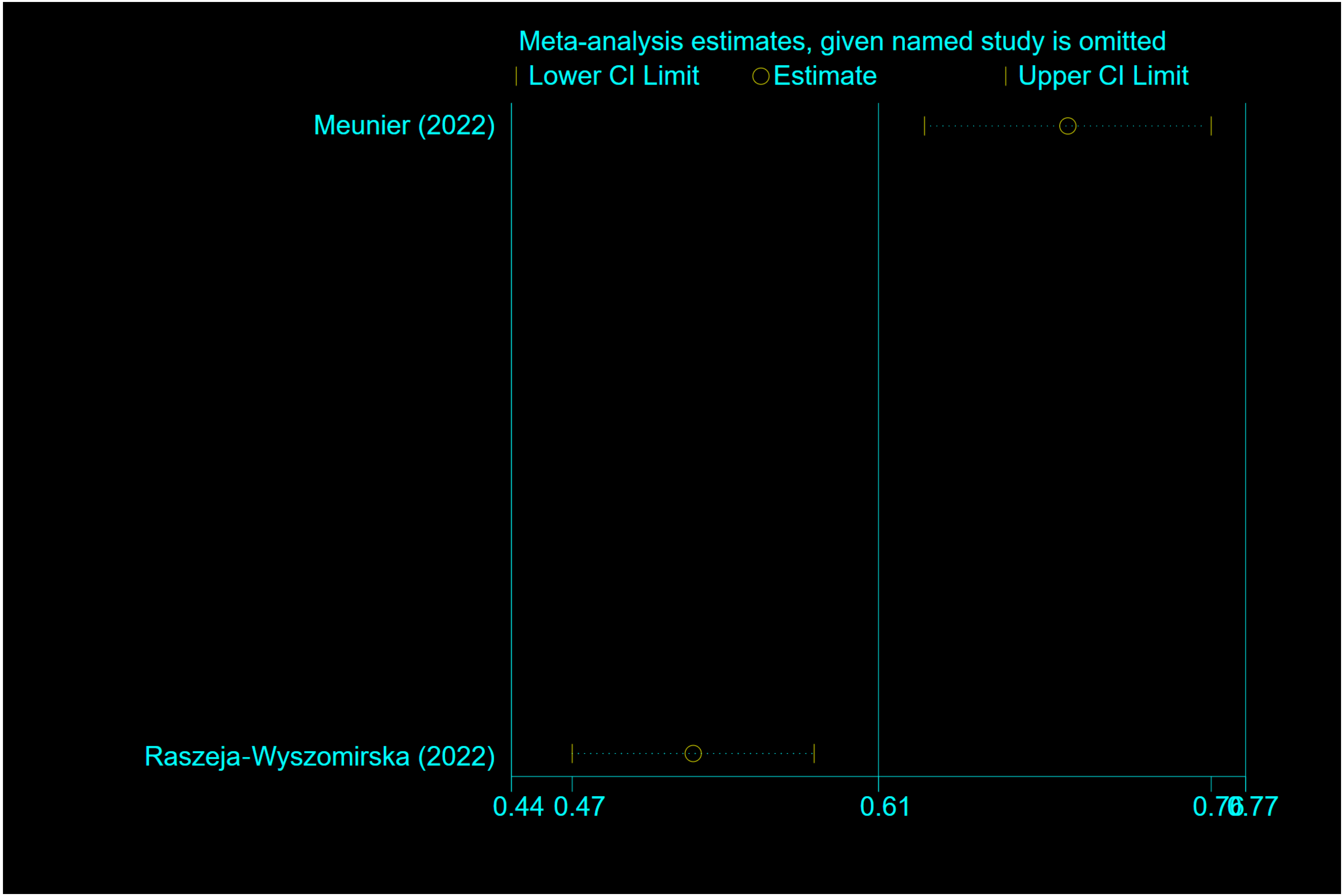

Meta-analysis estimates, given named study is omitted
Lower CI Limit
Estimate
Upper CI Limit
Meunier (2022)
Raszeja-Wyszomirska (2022)
0.44
0.47
0.61
0.76
0.77
