# Supplementary material for: Factors influencing immunogenicity and safety of SARS-CoV-2 vaccine in liver transplantation recipients: a systematic review and meta-analysis
Source: Front Immunol. 2023 Sep 5;14:1145081. doi: 10.3389/fimmu.2023.1145081 (PMC10508849; doi:10.3389/fimmu.2023.1145081)
Supplement: Supplementary Figure 3 — Presence of leukopenia on seroconversion rate. [file Image_3.pdf]

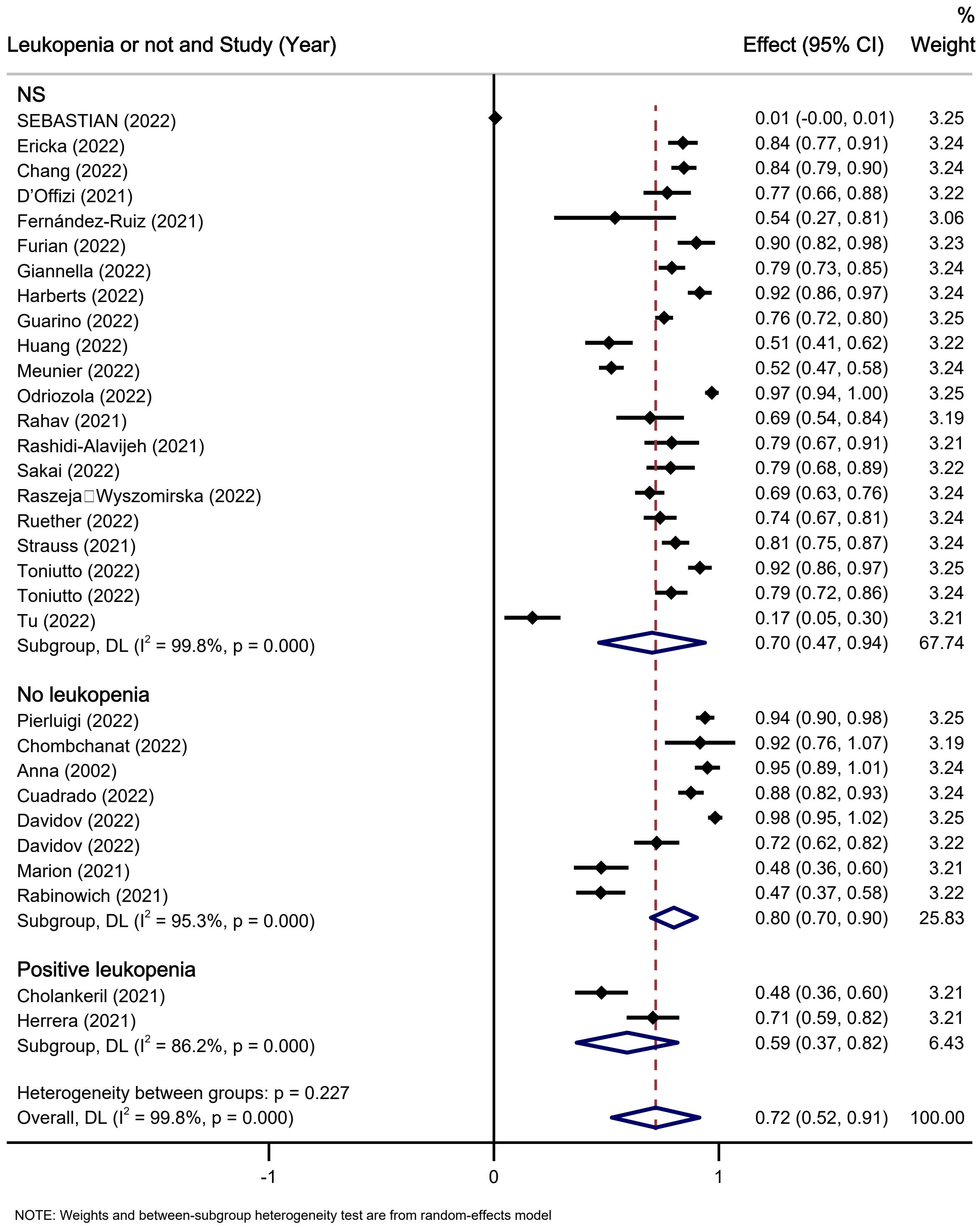

Leukopenia or not and Study (Year)
Effect (95% CI)
% Weight
NS
SEBASTIAN (2022) 0.01 (-0.00, 0.01) 3.25
Ericka (2022) 0.84 (0.77, 0.91) 3.24
Chang (2022) 0.84 (0.79, 0.90) 3.24
D’Offizi (2021) 0.77 (0.66, 0.88) 3.22
Fernández-Ruiz (2021) 0.54 (0.27, 0.81) 3.06
Furian (2022) 0.90 (0.82, 0.98) 3.23
Giannella (2022) 0.79 (0.73, 0.85) 3.24
Harberts (2022) 0.92 (0.86, 0.97) 3.24
Guarino (2022) 0.76 (0.72, 0.80) 3.25
Huang (2022) 0.51 (0.41, 0.62) 3.22
Meunier (2022) 0.52 (0.47, 0.58) 3.24
Odriozola (2022) 0.97 (0.94, 1.00) 3.25
Rahav (2021) 0.69 (0.54, 0.84) 3.19
Rashidi-Alavijeh (2021) 0.79 (0.67, 0.91) 3.21
Sakai (2022) 0.79 (0.68, 0.89) 3.22
Raszeja□Wyszomirska (2022) 0.69 (0.63, 0.76) 3.24
Ruether (2022) 0.74 (0.67, 0.81) 3.24
Strauss (2021) 0.81 (0.75, 0.87) 3.24
Toniutto (2022) 0.92 (0.86, 0.97) 3.25
Toniutto (2022) 0.79 (0.72, 0.86) 3.24
Tu (2022) 0.17 (0.05, 0.30) 3.21
Subgroup, DL (I^2 = 99.8%, p = 0.000) 0.70 (0.47, 0.94) 67.74
No leukopenia
Pierluigi (2022) 0.94 (0.90, 0.98) 3.25
Chombchanat (2022) 0.92 (0.76, 1.07) 3.19
Anna (2002) 0.95 (0.89, 1.01) 3.24
Cuadrado (2022) 0.88 (0.82, 0.93) 3.24
Davidov (2022) 0.98 (0.95, 1.02) 3.25
Davidov (2022) 0.72 (0.62, 0.82) 3.22
Marion (2021) 0.48 (0.36, 0.60) 3.21
Rabinowich (2021) 0.47 (0.37, 0.58) 3.22
Subgroup, DL (I^2 = 95.3%, p = 0.000) 0.80 (0.70, 0.90) 25.83
Positive leukopenia
Cholankeril (2021) 0.48 (0.36, 0.60) 3.21
Herrera (2021) 0.71 (0.59, 0.82) 3.21
Subgroup, DL (I^2 = 86.2%, p = 0.000) 0.59 (0.37, 0.82) 6.43
Heterogeneity between groups: p = 0.227
Overall, DL (I^2 = 99.8%, p = 0.000) 0.72 (0.52, 0.91) 100.00
-1
0
1
NOTE: Weights and between-subgroup heterogeneity test are from random-effects model

Study omitted	Estimate	[95% Conf.	Interval]
Pierluigi (2022)	.77649826	.64604211	.90695435
Chombchanat (2022)	.78653306	.67696136	.89610469
Anna (2002)	.77624661	.65578341	.8967098
Cuadrado (2022)	.78740054	.66730201	.90749913
Davidov (2022)	.76999533	.64605105	.89393961
Davidov (2022)	.81141955	.70407343	.91876566
Marion (2021)	.84528297	.75434589	.93622011
Rabinowich (2021)	.84889817	.76315051	.93464583
Combined	.80046652	.69841508	.90251797

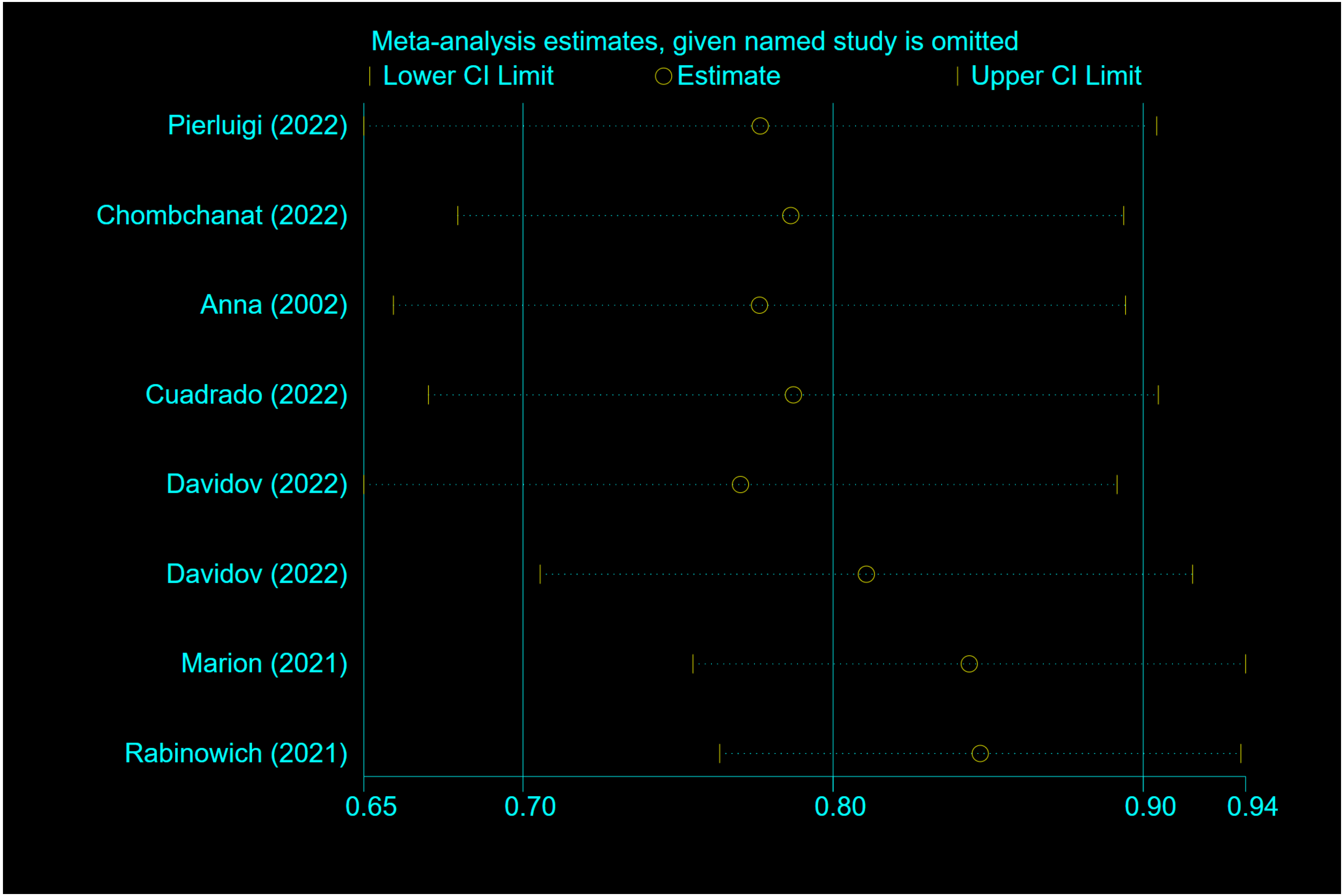
Meta-analysis estimates, given named study is omitted
Lower CI Limit
Estimate
Upper CI Limit
Pierluigi (2022)
Chombchanat (2022)
Anna (2002)
Cuadrado (2022)
Davidov (2022)
Davidov (2022)
Marion (2021)
Rabinowich (2021)
0.65
0.70
0.80
0.90
0.94

Study omitted	Estimate	[95% Conf.	Interval]
**Cholankeril (2021)**	**.70689648**	**.58975178**	**.82404119**
**Herrera (2021)**	**.4782609**	**.3603965**	**.5961253**
**Combined**	**.59267498**	**.36861631**	**.81673365**

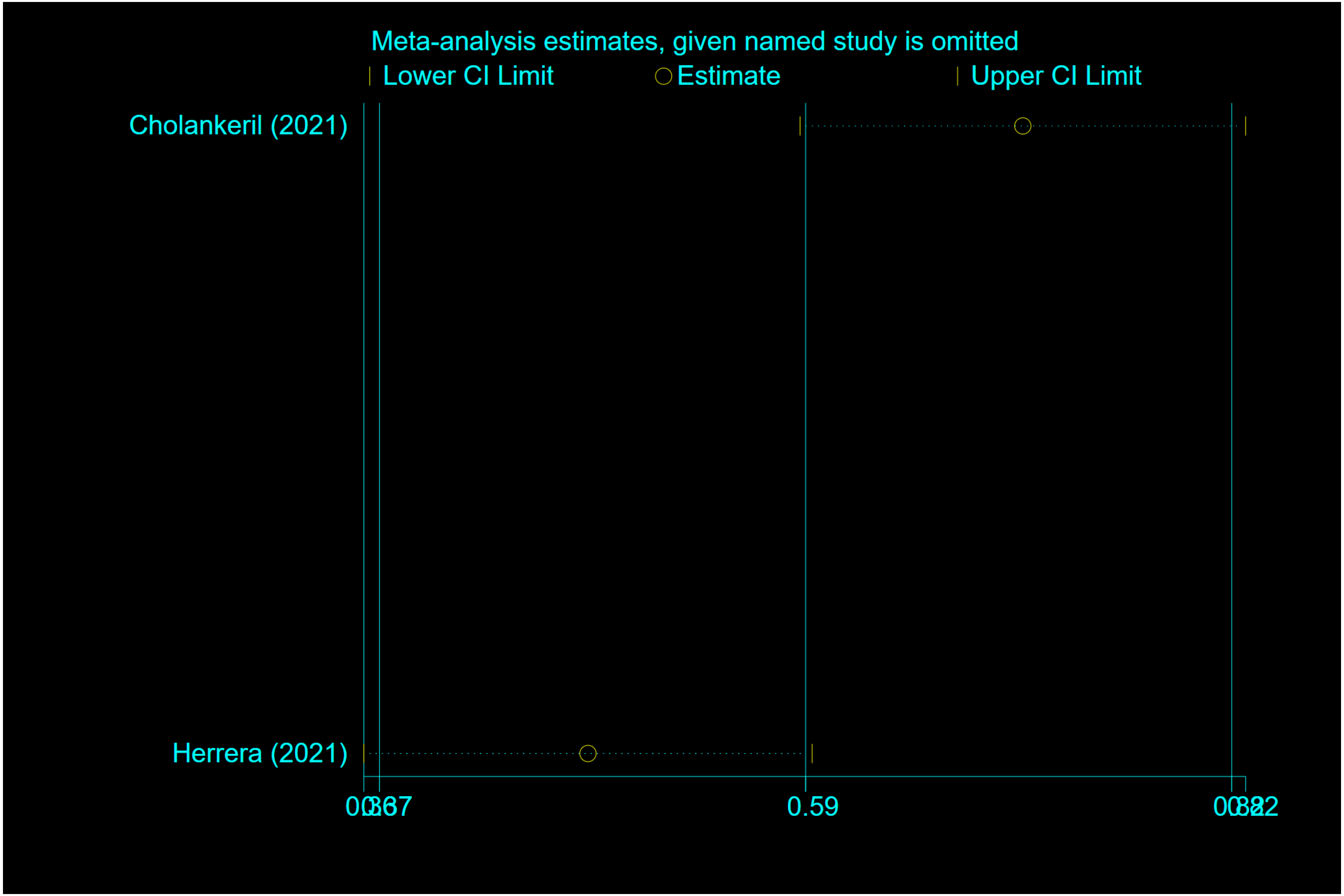
Meta-analysis estimates, given named study is omitted
Lower CI Limit
Estimate
Upper CI Limit
Cholankeril (2021)
Herrera (2021)
0.59
