# Supplementary material for: Factors influencing immunogenicity and safety of SARS-CoV-2 vaccine in liver transplantation recipients: a systematic review and meta-analysis
Source: Front Immunol. 2023 Sep 5;14:1145081. doi: 10.3389/fimmu.2023.1145081 (PMC10508849; doi:10.3389/fimmu.2023.1145081)
Supplement: Supplementary file 5 [file DataSheet_2.pdf]

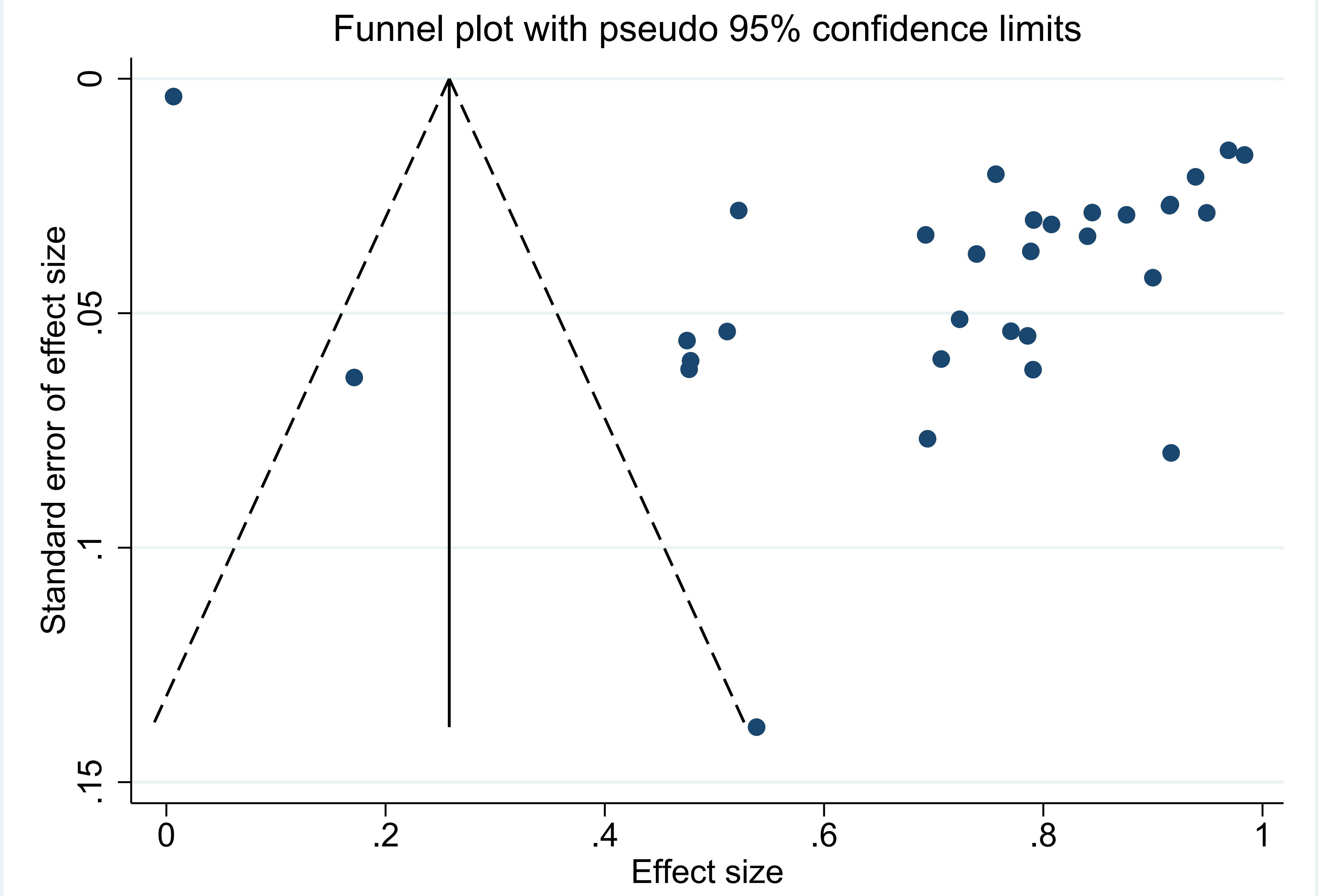
Funnel plot with pseudo 95% confidence limits
Standard error of effect size
0
.05
.1
.15
Effect size
0
.2
.4
.6
.8
1

Number of studies = 31 Root MSE = 15.56

Std_Eff	Coefficient	Std. err.	t	P>|t|	[95% conf.	interval]
slope	.022389	.0639876	0.35	0.729	-.1084804	.1532584
bias	20.99878	3.61517	5.81	0.000	13.60492	28.39263

Test of H0: no small-study effects P = 0.000
