# Supplementary material for: Factors influencing immunogenicity and safety of SARS-CoV-2 vaccine in liver transplantation recipients: a systematic review and meta-analysis
Source: Front Immunol. 2023 Sep 5;14:1145081. doi: 10.3389/fimmu.2023.1145081 (PMC10508849; doi:10.3389/fimmu.2023.1145081)
Supplement: Supplementary file 6 [file DataSheet_3.pdf]

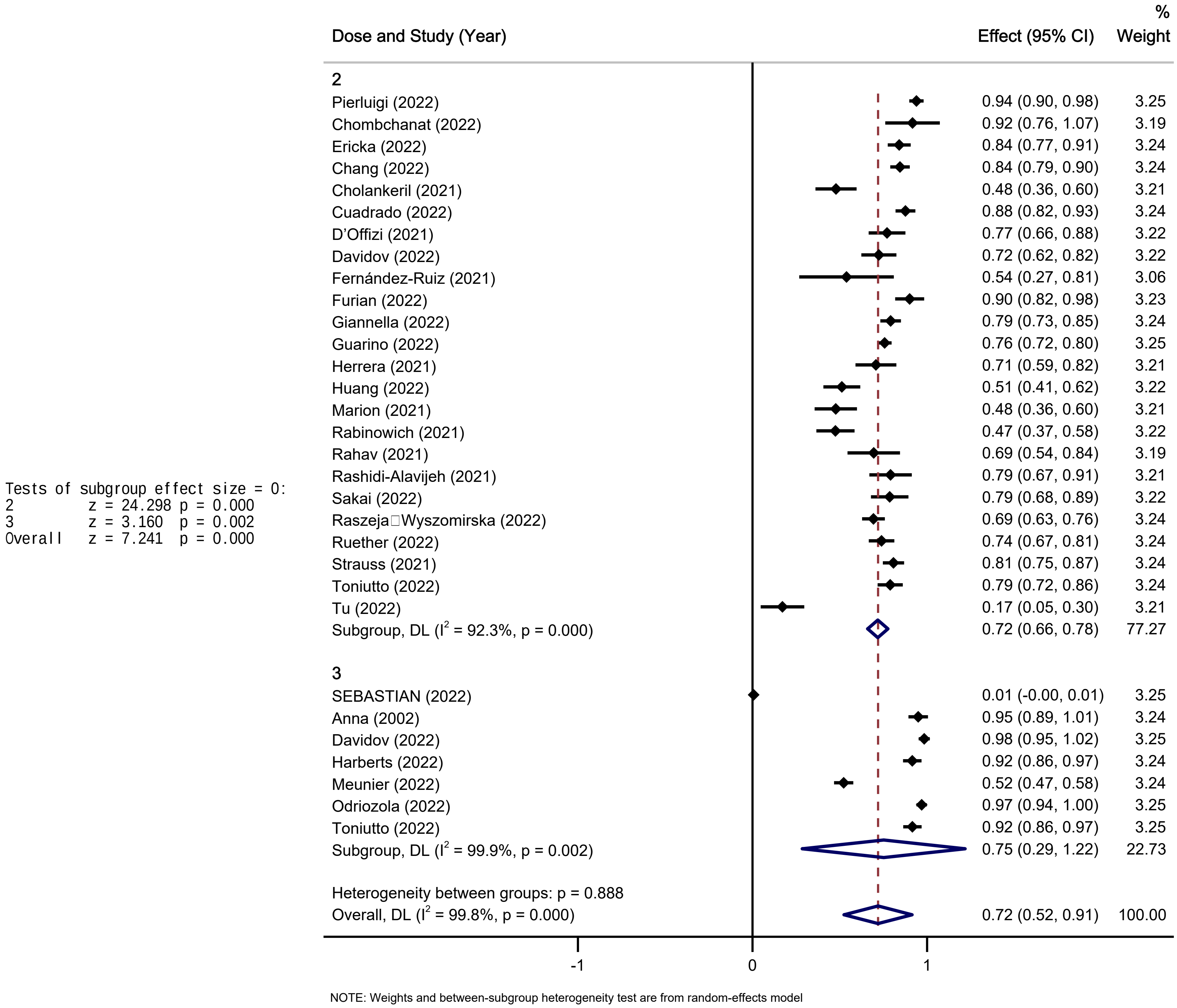
Dose and Study (Year)
Effect (95% CI)
% Weight
2
Pierluigi (2022) 0.94 (0.90, 0.98) 3.25
Chombchanat (2022) 0.92 (0.76, 1.07) 3.19
Ericka (2022) 0.84 (0.77, 0.91) 3.24
Chang (2022) 0.84 (0.79, 0.90) 3.24
Cholankeril (2021) 0.48 (0.36, 0.60) 3.21
Cuadrado (2022) 0.88 (0.82, 0.93) 3.24
D'Offizi (2021) 0.77 (0.66, 0.88) 3.22
Davidov (2022) 0.72 (0.62, 0.82) 3.22
Fernández-Ruiz (2021) 0.54 (0.27, 0.81) 3.06
Furian (2022) 0.90 (0.82, 0.98) 3.23
Giannella (2022) 0.79 (0.73, 0.85) 3.24
Guarino (2022) 0.76 (0.72, 0.80) 3.25
Herrera (2021) 0.71 (0.59, 0.82) 3.21
Huang (2022) 0.51 (0.41, 0.62) 3.22
Marion (2021) 0.48 (0.36, 0.60) 3.21
Rabinowich (2021) 0.47 (0.37, 0.58) 3.22
Rahav (2021) 0.69 (0.54, 0.84) 3.19
Rashidi-Alavijeh (2021) 0.79 (0.67, 0.91) 3.21
Sakai (2022) 0.79 (0.68, 0.89) 3.22
Raszeja□Wyszomirska (2022) 0.69 (0.63, 0.76) 3.24
Ruether (2022) 0.74 (0.67, 0.81) 3.24
Strauss (2021) 0.81 (0.75, 0.87) 3.24
Toniutto (2022) 0.79 (0.72, 0.86) 3.24
Tu (2022) 0.17 (0.05, 0.30) 3.21
Subgroup, DL (I^2 = 92.3%, p = 0.000) 0.72 (0.66, 0.78) 77.27
3
SEBASTIAN (2022) 0.01 (-0.00, 0.01) 3.25
Anna (2002) 0.95 (0.89, 1.01) 3.24
Davidov (2022) 0.98 (0.95, 1.02) 3.25
Harberts (2022) 0.92 (0.86, 0.97) 3.24
Meunier (2022) 0.52 (0.47, 0.58) 3.24
Odriozola (2022) 0.97 (0.94, 1.00) 3.25
Toniutto (2022) 0.92 (0.86, 0.97) 3.25
Subgroup, DL (I^2 = 99.9%, p = 0.002) 0.75 (0.29, 1.22) 22.73
Heterogeneity between groups: p = 0.888
Overall, DL (I^2 = 99.8%, p = 0.000) 0.72 (0.52, 0.91) 100.00
-1
0
1
Tests of subgroup effect size = 0:
2 z = 24.298 p = 0.000
3 z = 3.160 p = 0.002
Overall z = 7.241 p = 0.000
NOTE: Weights and between-subgroup heterogeneity test are from random-effects model

Study omitted	Estimate	[95% Conf.	Interval]
Pierluigi (2022)	.70749992	.65122074	.76377904
Chombchanat (2022)	.71027565	.65122509	.76932615
Ericka (2022)	.71149319	.65085769	.77212876
Chang (2022)	.71109474	.65003461	.77215493
Cholankeril (2021)	.72809827	.67090797	.78528857
Cuadrado (2022)	.70976758	.64939374	.77014142
D’Offizi (2021)	.71516627	.65531665	.77501589
Davidov (2022)	.71719676	.65738326	.7770102
Fernández-Ruiz (2021)	.72194684	.66346961	.780424
Furian (2022)	.70923471	.64971483	.7687546
Giannella (2022)	.71360189	.6522513	.77495247
Guarino (2022)	.71471786	.65124083	.7781949
Herrera (2021)	.71796501	.65841794	.77751201
Huang (2022)	.72699338	.66961491	.78437185
Marion (2021)	.7280159	.67073798	.78529382
Rabinowich (2021)	.72860974	.67179632	.7854231
Rahav (2021)	.71843529	.65918291	.77768767
Rashidi-Alavijeh (2021)	.7144739	.65483451	.77411336
Sakai (2022)	.71452963	.65470654	.77435279
Raszeja?Wyszomirska (2022)	.71854639	.65827781	.77881497
Ruether (2022)	.7163052	.65580046	.77680987
Strauss (2021)	.7128846	.65172178	.77404743
Toniutto (2022)	.71395797	.65329027	.77462566
Tu (2022)	.74226177	.69227028	.79225332
Combined	.71763771	.65974932	.77552611

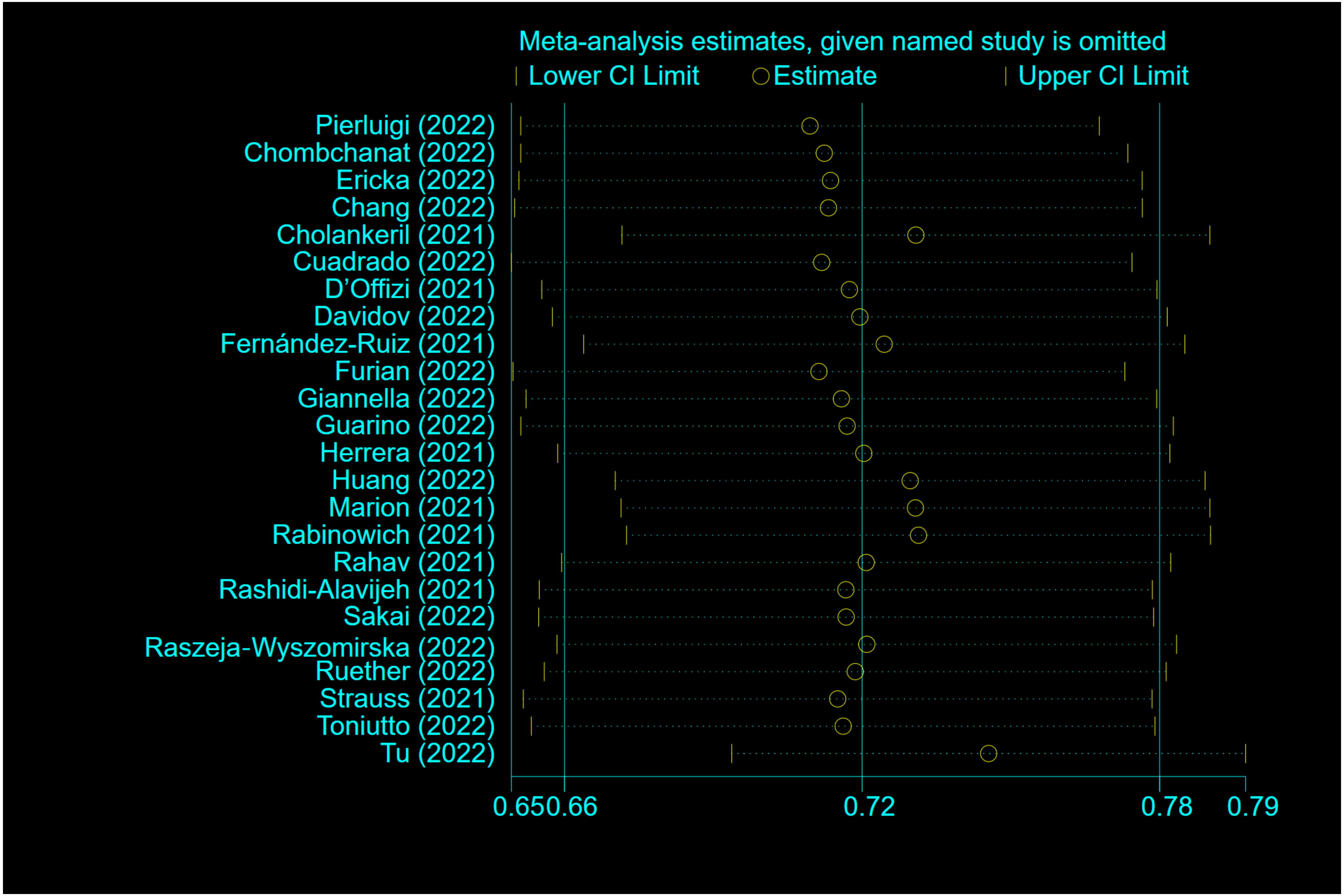

Meta-analysis estimates, given named study is omitted
Lower CI Limit
Estimate
Upper CI Limit
Pierluigi (2022)
Chombchanat (2022)
Ericka (2022)
Chang (2022)
Cholankeril (2021)
Cuadrado (2022)
D'Offizi (2021)
Davidov (2022)
Fernández-Ruiz (2021)
Furian (2022)
Giannella (2022)
Guarino (2022)
Herrera (2021)
Huang (2022)
Marion (2021)
Rabinowich (2021)
Rahav (2021)
Rashidi-Alavijeh (2021)
Sakai (2022)
Raszeja-Wyszomirska (2022)
Ruether (2022)
Strauss (2021)
Toniutto (2022)
Tu (2022)
0.65
0.66
0.72
0.78
0.79

Study omitted	Estimate	[95% Conf.	Interval]
SEBASTIAN (2022)	.87666094	.75780594	.99551594
Anna (2002)	.71861911	.2140017	1.2232366
Davidov (2022)	.71281374	.21635228	1.2092752
Harberts (2022)	.72428459	.21741906	1.2311502
Meunier (2022)	.78973621	.26685631	1.3126161
Odriozola (2022)	.71524912	.21687929	1.213619
Toniutto (2022)	.72415137	.21727574	1.2310271
Combined	.75152645	.28538273	1.2176702

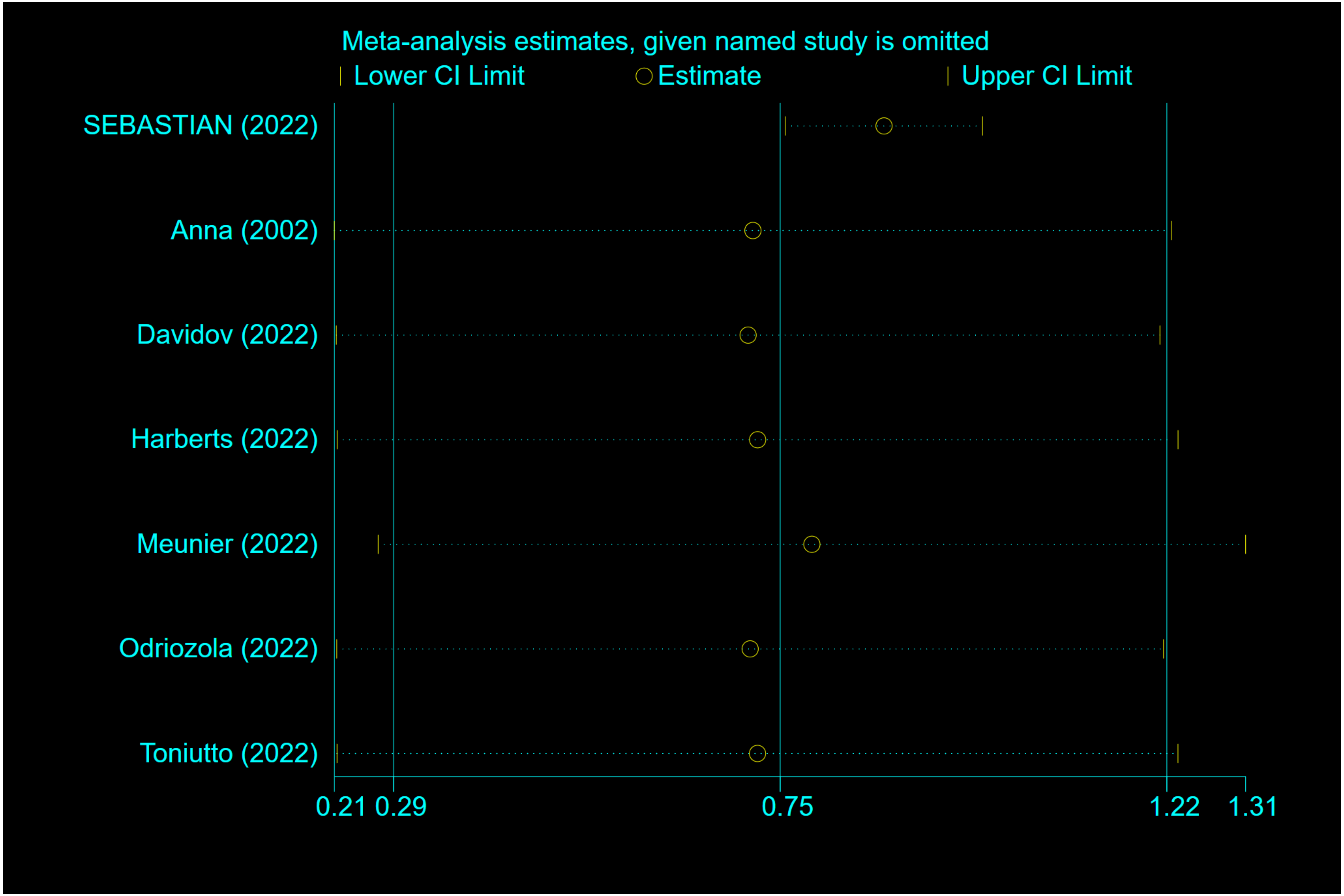
Meta-analysis estimates, given named study is omitted
Lower CI Limit
Estimate
Upper CI Limit
SEBASTIAN (2022)
Anna (2002)
Davidov (2022)
Harberts (2022)
Meunier (2022)
Odriozola (2022)
Toniutto (2022)
0.21
0.29
0.75
1.22
1.31
