# Supplementary material for: Factors influencing immunogenicity and safety of SARS-CoV-2 vaccine in liver transplantation recipients: a systematic review and meta-analysis
Source: Front Immunol. 2023 Sep 5;14:1145081. doi: 10.3389/fimmu.2023.1145081 (PMC10508849; doi:10.3389/fimmu.2023.1145081)
Supplement: Supplementary file 7 [file DataSheet_4.pdf]

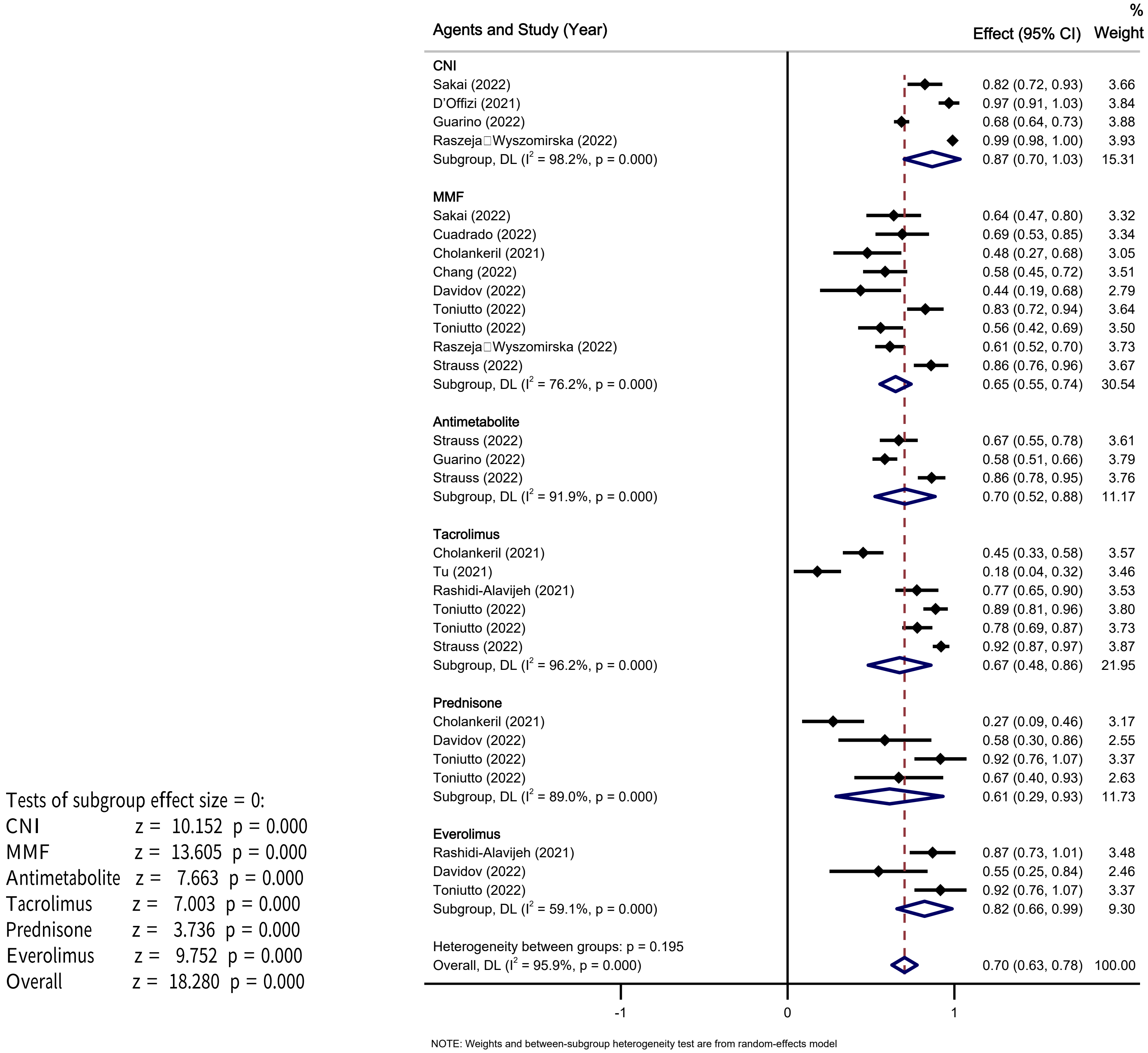
Agents and Study (Year)
Effect (95% CI)
% Weight
CNI
Sakai (2022) 0.82 (0.72, 0.93) 3.66
D'Offizi (2021) 0.97 (0.91, 1.03) 3.84
Guarino (2022) 0.68 (0.64, 0.73) 3.88
Raszeja□Wyszomirska (2022) 0.99 (0.98, 1.00) 3.93
Subgroup, DL (I^2 = 98.2%, p = 0.000) 0.87 (0.70, 1.03) 15.31
MMF
Sakai (2022) 0.64 (0.47, 0.80) 3.32
Cuadrado (2022) 0.69 (0.53, 0.85) 3.34
Cholankeril (2021) 0.48 (0.27, 0.68) 3.05
Chang (2022) 0.58 (0.45, 0.72) 3.51
Davidov (2022) 0.44 (0.19, 0.68) 2.79
Toniutto (2022) 0.83 (0.72, 0.94) 3.64
Toniutto (2022) 0.56 (0.42, 0.69) 3.50
Raszeja□Wyszomirska (2022) 0.61 (0.52, 0.70) 3.73
Strauss (2022) 0.86 (0.76, 0.96) 3.67
Subgroup, DL (I^2 = 76.2%, p = 0.000) 0.65 (0.55, 0.74) 30.54
Antimetabolite
Strauss (2022) 0.67 (0.55, 0.78) 3.61
Guarino (2022) 0.58 (0.51, 0.66) 3.79
Strauss (2022) 0.86 (0.78, 0.95) 3.76
Subgroup, DL (I^2 = 91.9%, p = 0.000) 0.70 (0.52, 0.88) 11.17
Tacrolimus
Cholankeril (2021) 0.45 (0.33, 0.58) 3.57
Tu (2021) 0.18 (0.04, 0.32) 3.46
Rashidi-Alavijeh (2021) 0.77 (0.65, 0.90) 3.53
Toniutto (2022) 0.89 (0.81, 0.96) 3.80
Toniutto (2022) 0.78 (0.69, 0.87) 3.73
Strauss (2022) 0.92 (0.87, 0.97) 3.87
Subgroup, DL (I^2 = 96.2%, p = 0.000) 0.67 (0.48, 0.86) 21.95
Prednisone
Cholankeril (2021) 0.27 (0.09, 0.46) 3.17
Davidov (2022) 0.58 (0.30, 0.86) 2.55
Toniutto (2022) 0.92 (0.76, 1.07) 3.37
Toniutto (2022) 0.67 (0.40, 0.93) 2.63
Subgroup, DL (I^2 = 89.0%, p = 0.000) 0.61 (0.29, 0.93) 11.73
Everolimus
Rashidi-Alavijeh (2021) 0.87 (0.73, 1.01) 3.48
Davidov (2022) 0.55 (0.25, 0.84) 2.46
Toniutto (2022) 0.92 (0.76, 1.07) 3.37
Subgroup, DL (I^2 = 59.1%, p = 0.000) 0.82 (0.66, 0.99) 9.30
Heterogeneity between groups: p = 0.195
Overall, DL (I^2 = 95.9%, p = 0.000) 0.70 (0.63, 0.78) 100.00
-1
0
1
NOTE: Weights and between-subgroup heterogeneity test are from random-effects model
Tests of subgroup effect size = 0:
CNI z = 10.152 p = 0.000
MMF z = 13.605 p = 0.000
Antimetabolite z = 7.663 p = 0.000
Tacrolimus z = 7.003 p = 0.000
Prednisone z = 3.736 p = 0.000
Everolimus z = 9.752 p = 0.000
Overall z = 18.280 p = 0.000

Study omitted	Estimate	[95% Conf.	Interval]
Davidov (2022)	.86704916	.69966209	1.0344362
Raszeja-Wyszomirska (2022)	.82421112	.6283229	1.0200994
Guarino (2022)	.94405127	.87162733	1.0164753
D’Offizi (2021)	.83302259	.60216677	1.0638784
Sakai (2022)	.88045448	.68158132	1.0793277
Combined	.86704918	.69966208	1.0344363

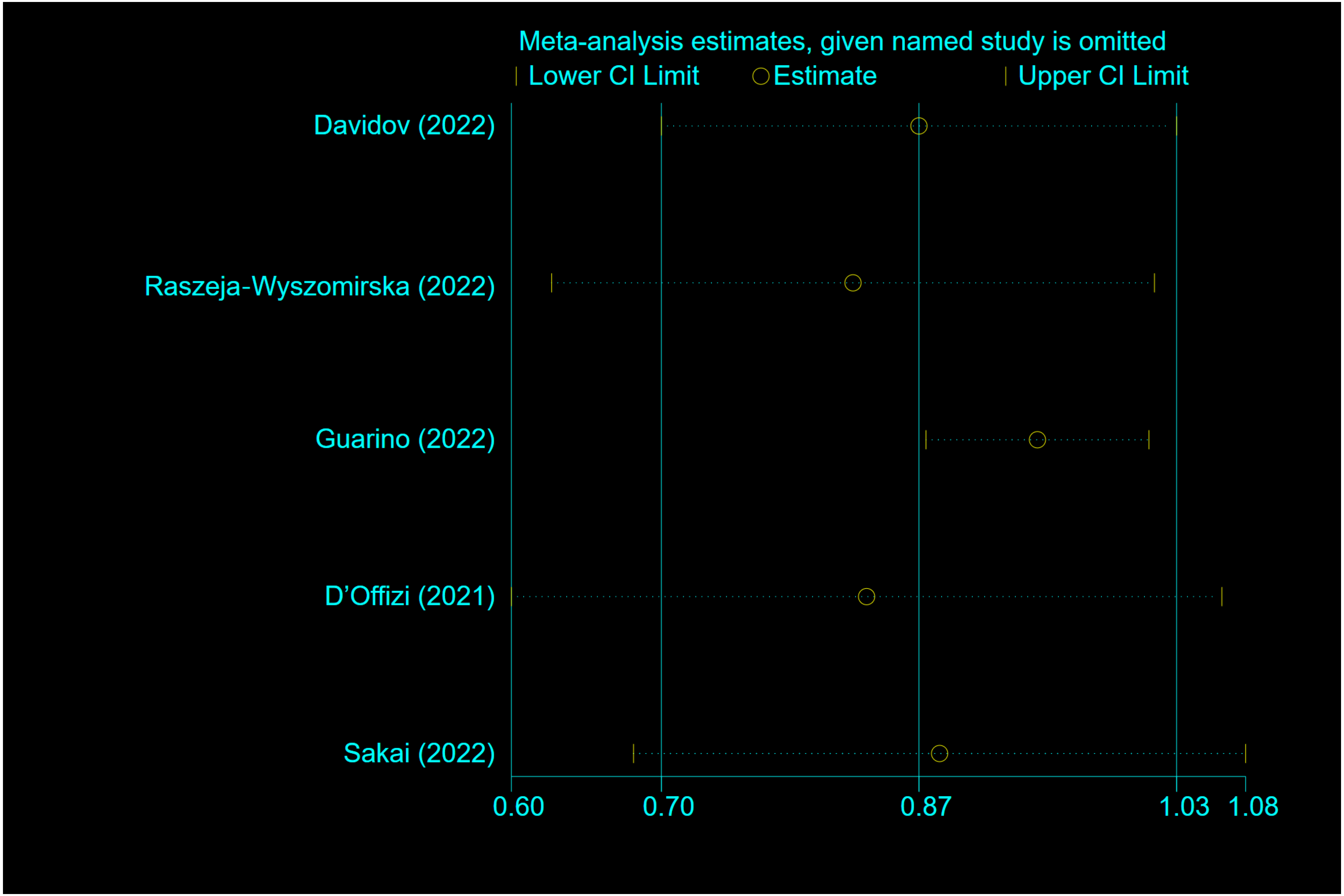
Meta-analysis estimates, given named study is omitted
Lower CI Limit
Estimate
Upper CI Limit
Davidov (2022)
Raszeja-Wyszomirska (2022)
Guarino (2022)
D’Offizi (2021)
Sakai (2022)
0.60
0.70
0.87
1.03
1.08

Study omitted	Estimate	[95% Conf.	Interval]
Raszeja Wyszomirska (2022)	.65031236	.54148102	.75914371
Strauss (2022)	.62049335	.5372321	.70375466
Toniutto (2022)	.62302876	.52818513	.71787232
Chang (2022)	.65493327	.55191612	.75795048
Toniutto (2022)	.65910321	.55835396	.75985247
Cuadrado (2022)	.64145732	.53761464	.74529994
Sakai (2022)	.64767754	.54442543	.75092959
Cholankeril (2021)	.66463232	.56893015	.76033449
Davidov (2022)	.66528809	.57098287	.75959325
Combined	.64769058	.55438636	.74099481

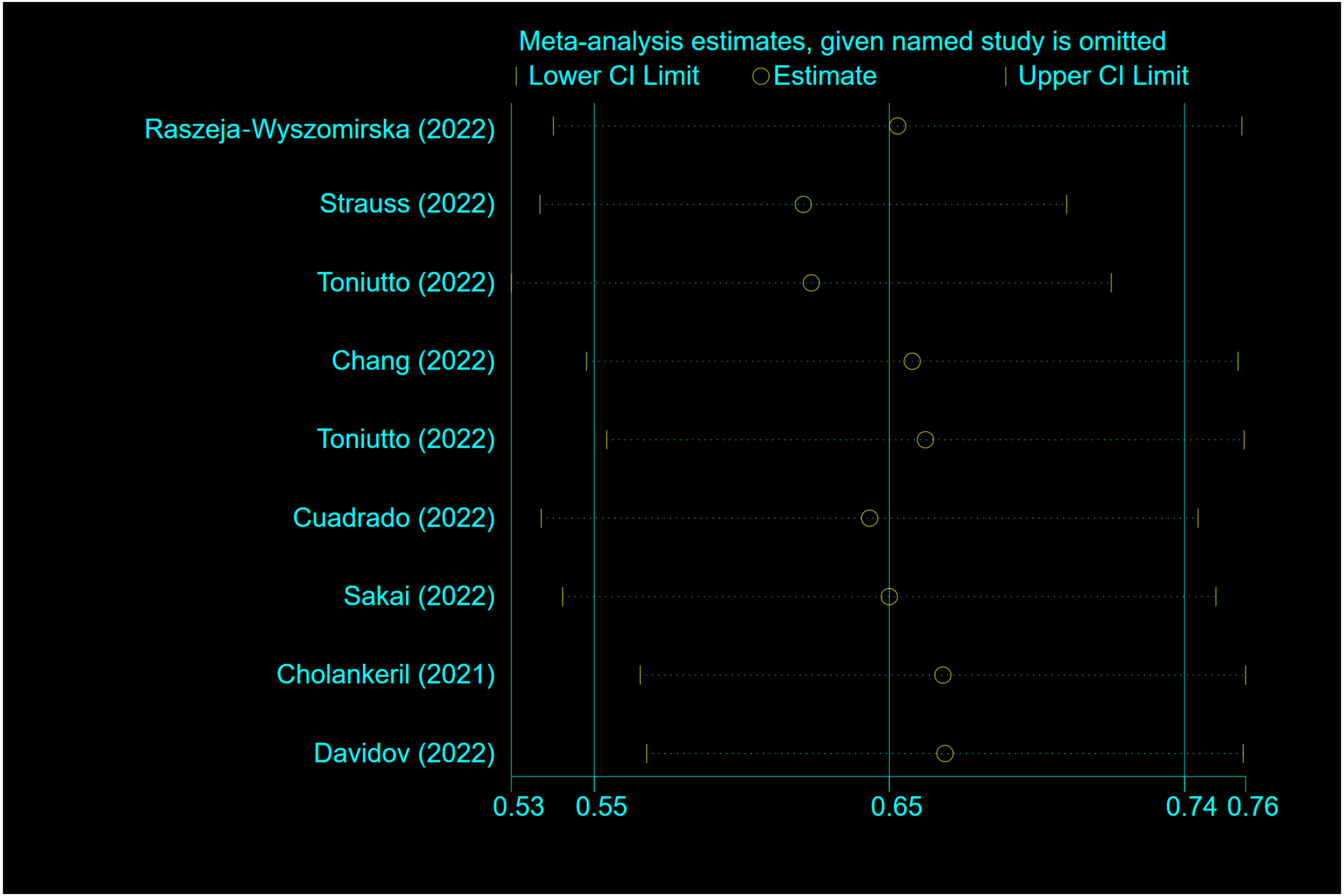

Meta-analysis estimates, given named study is omitted
Lower CI Limit
Estimate
Upper CI Limit
Raszeja-Wyszomirska (2022)
Strauss (2022)
Toniutto (2022)
Chang (2022)
Toniutto (2022)
Cuadrado (2022)
Sakai (2022)
Cholankeril (2021)
Davidov (2022)
0.53
0.55
0.65
0.74
0.76

Study omitted	Estimate	[95% Conf.	Interval]
Guarino (2022)	.76916897	.57630289	.96203506
Strauss (2022)	.61347854	.53499746	.69195956
Strauss (2022)	.72288263	.44819319	.99757206
Combined	.70474726	.5245027	.88499182

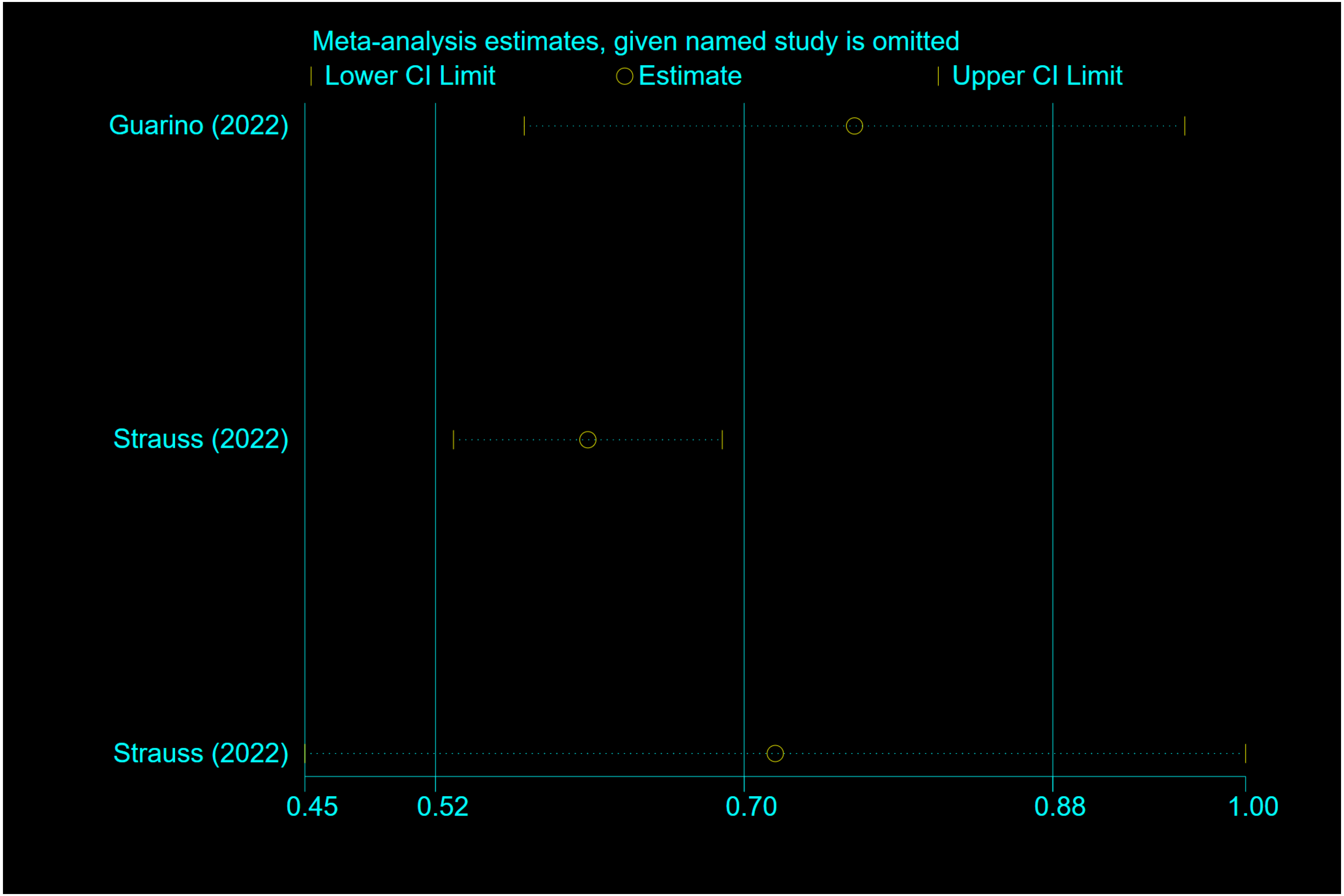

Meta-analysis estimates, given named study is omitted
Lower CI Limit
Estimate
Upper CI Limit
Guarino (2022)
Strauss (2022)
Strauss (2022)
0.45
0.52
0.70
0.88
1.00

Study omitted	Estimate	[95% Conf.	Interval]
Strauss (2022)	.61856115	.38641652	.85070574
Toniutto (2022)	.62557316	.38229081	.86885554
Toniutto (2022)	.64872611	.41550183	.88195032
Cholankeril (2021)	.71592396	.52625203	.90559596
Rashidi-Alavijeh (2021)	.65093708	.43243572	.86943841
Tu (2021)	.76952922	.63205659	.90700179
Combined	.67189076	.48384292	.85993859

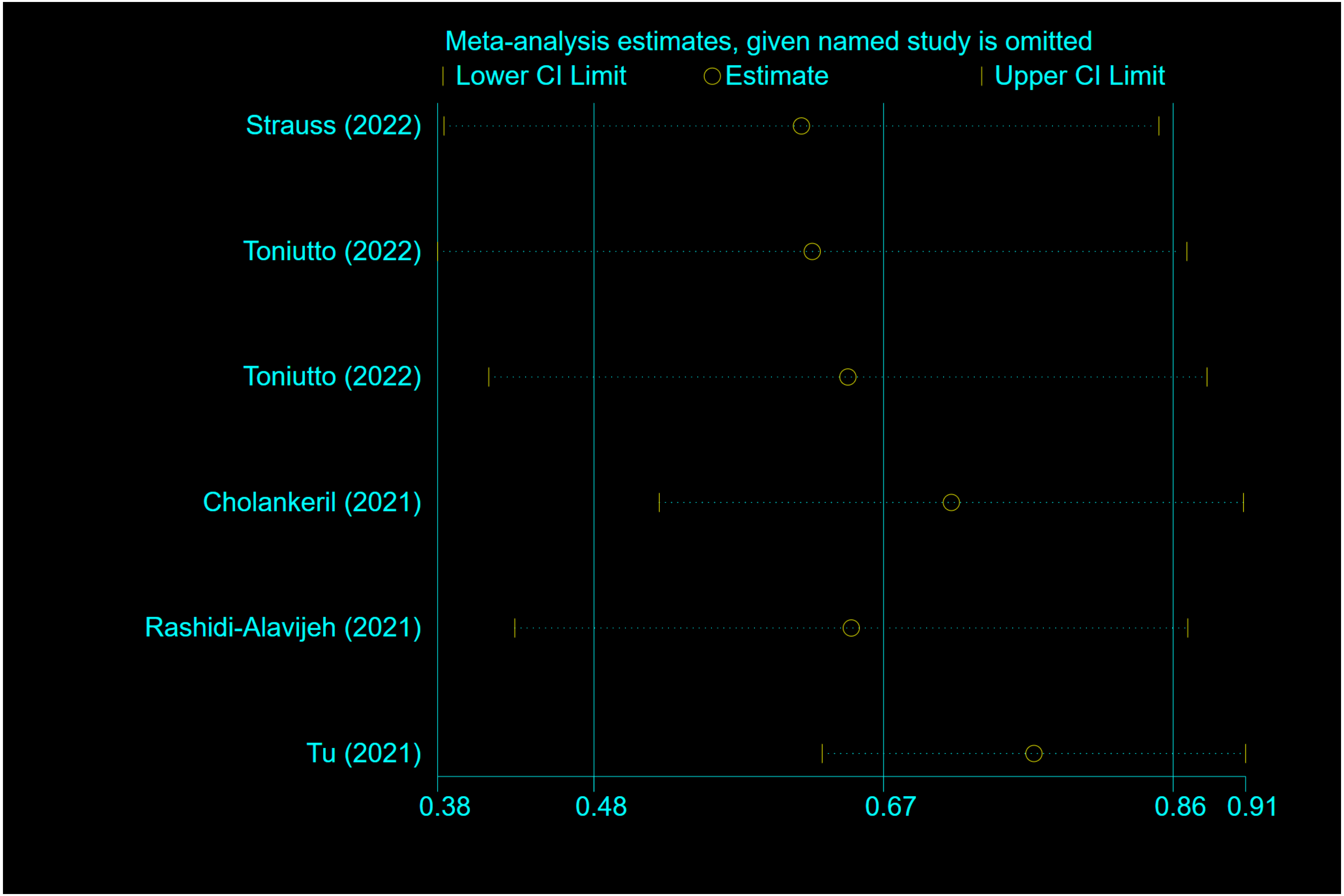
Meta-analysis estimates, given named study is omitted
Lower CI Limit
Estimate
Upper CI Limit
Strauss (2022)
Toniutto (2022)
Toniutto (2022)
Cholankeril (2021)
Rashidi-Alavijeh (2021)
Tu (2021)
0.38
0.48
0.67
0.86
0.91

Study omitted	Estimate	[95% Conf.	Interval]
Toniutto (2022)	.49160609	.2323011	.75091106
Cholankeril (2021)	.74733716	.52831376	.96636063
Toniutto (2022)	.59324867	.16715103	1.0193462
Davidov (2022)	.61963803	.20057009	1.0387059
Combined	.61138741	.290613	.93216182

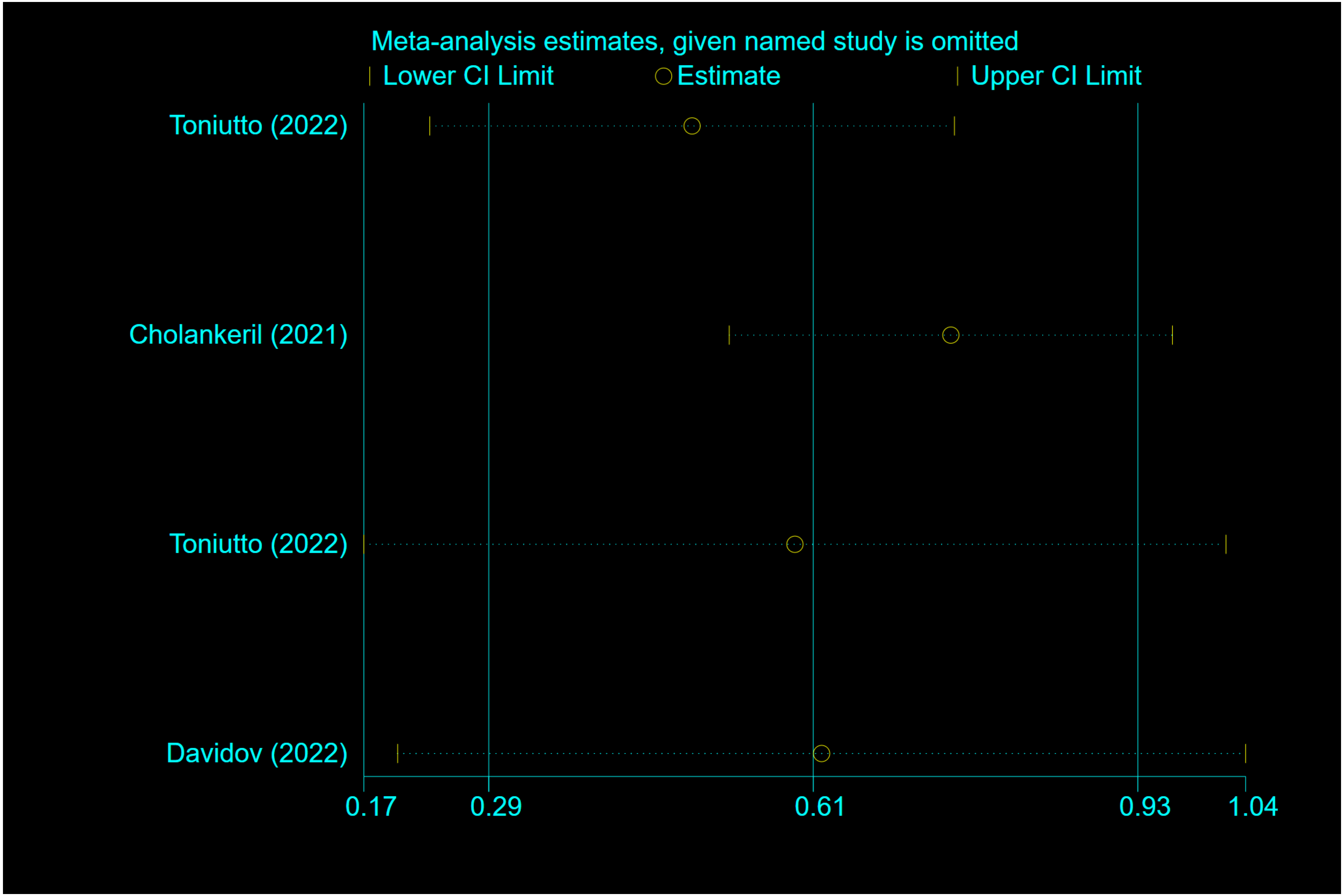

Meta-analysis estimates, given named study is omitted
Lower CI Limit
Estimate
Upper CI Limit
Toniutto (2022)
Cholankeril (2021)
Toniutto (2022)
Davidov (2022)
0.17
0.29
0.61
0.93
1.04

Study omitted	Estimate	[95% Conf. Interval]	
**Toniutto (2022)**	**.82138371**	**.65630078**	**.98646665**
**Rashidi-Alavijeh (2021)**	**.75284564**	**.39157894**	**1.1141124**
**Toniutto (2022)**	**.73467356**	**.42154488**	**1.0478023**
**Davidov (2022)**	**.89012575**	**.78680855**	**.99344295**
**Combined**	**.82138371**	**.65630079**	**.98646663**

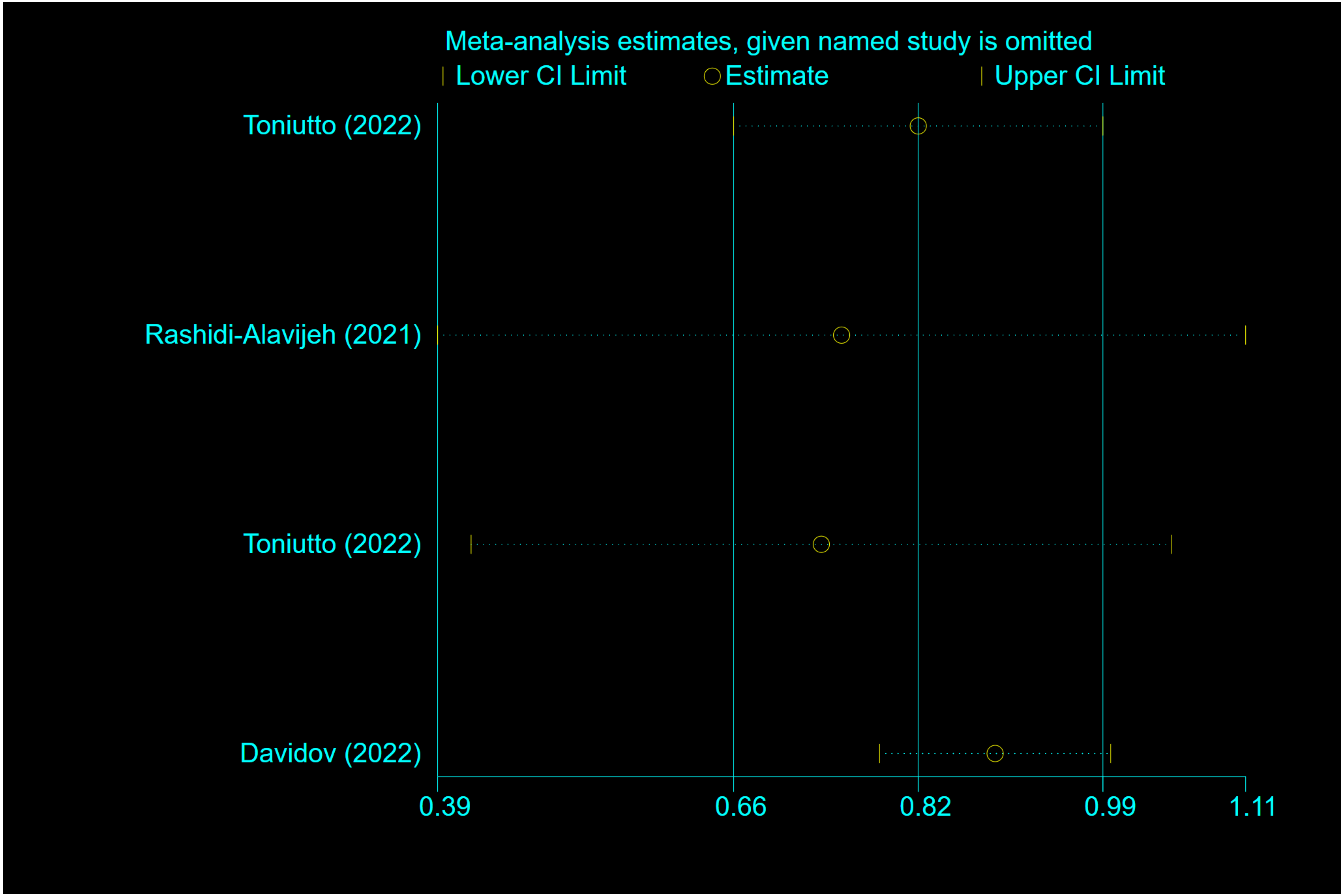

Meta-analysis estimates, given named study is omitted
Lower CI Limit
Estimate
Upper CI Limit
Toniutto (2022)
Rashidi-Alavijeh (2021)
Toniutto (2022)
Davidov (2022)
0.39
0.66
0.82
0.99
1.11
