# Supplementary material for: Factors influencing immunogenicity and safety of SARS-CoV-2 vaccine in liver transplantation recipients: a systematic review and meta-analysis
Source: Front Immunol. 2023 Sep 5;14:1145081. doi: 10.3389/fimmu.2023.1145081 (PMC10508849; doi:10.3389/fimmu.2023.1145081)
Supplement: Supplementary file 8 [file DataSheet_5.pdf]

Tests of subgroup effect size = 1:

>2 immunosuppressive drug	z = 0.362	p = 0.717
Age	z = 0.055	p = 0.956
Leukopenia	z = 1.114	p = 0.265
Low eGFR	z = 1.162	p = 0.245
MMF	z = 1.759	p = 0.079
Time since transplantati	z = 2.260	p = 0.024
Overall	z = 1.736	p = 0.083

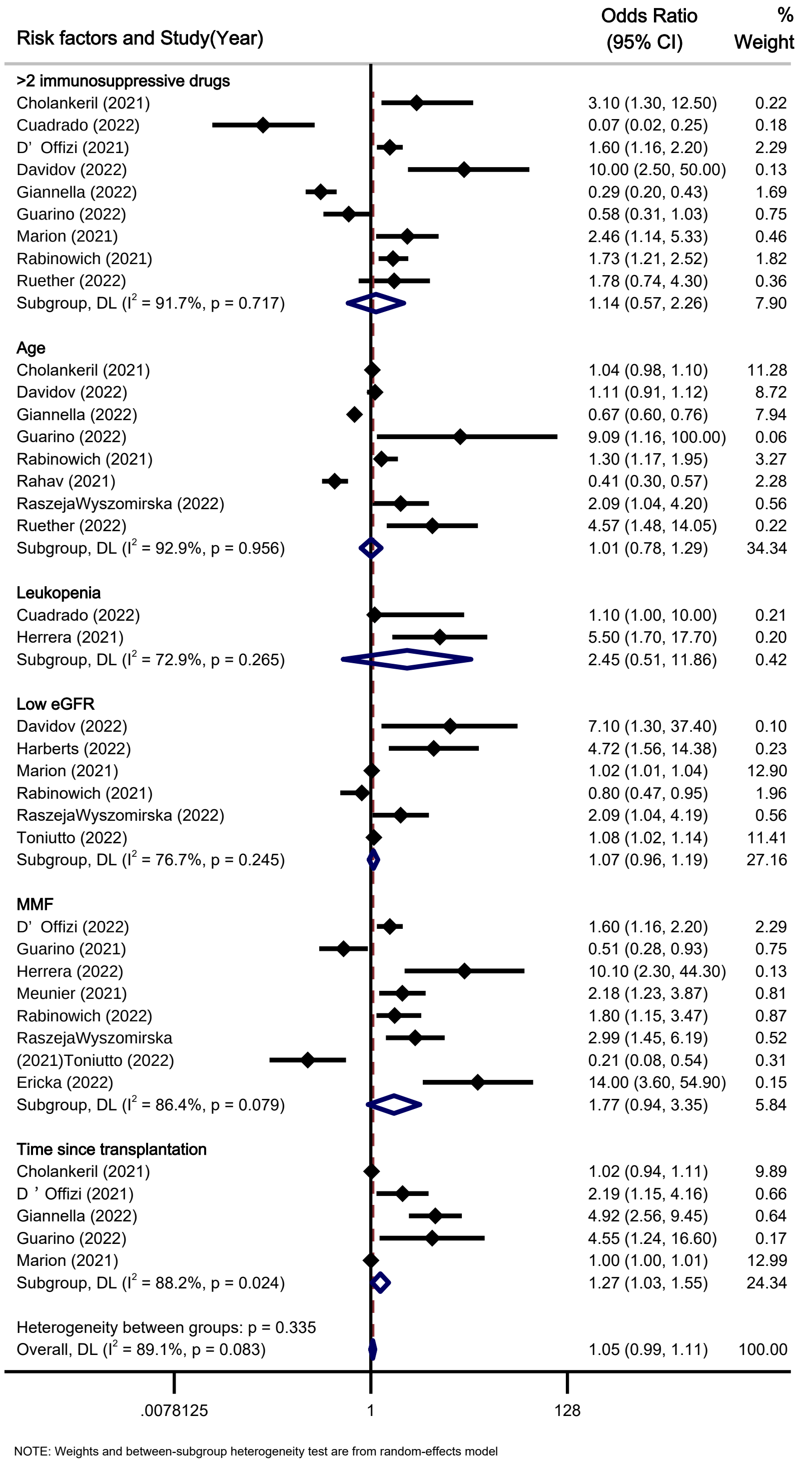

Study omitted	Estimate	[95% Conf.	Interval]
Cholankeril (2021)	1.0457373	.99143922	1.1030091
Cuadrado (2022)	1.0518378	.99804205	1.1085331
D’Offizi (2021)	1.0376058	.98341727	1.0947801
Davidov (2022)	1.0448096	.99089742	1.1016551
Giannella (2022)	1.0664326	1.0130153	1.1226667
Guarino (2022)	1.0529321	.99808025	1.1107986
Marion (2021)	1.0439749	.9897843	1.1011324
Rabinowich (2021)	1.0382277	.98415726	1.0952687
Ruether (2022)	1.0464245	.9919228	1.1039208
Cholankeril (2021)	1.050837	.9921416	1.1130049
Davidov (2022)	1.0429097	.98610777	1.1029836
Giannella (2022)	1.0849206	1.0289762	1.1439067
Guarino (2022)	1.0469301	.99261099	1.1042217
Rabinowich (2021)	1.040679	.98579174	1.0986223
Rahav (2021)	1.067816	1.0134668	1.1250798
Raszeja-Wyszomirska (2022)	1.0441374	.98985434	1.1013973
Ruether (2022)	1.0445491	.99050003	1.1015476
Cuadrado (2022)	1.0484543	.99379271	1.1061224
Herrera (2021)	1.0443197	.99035466	1.1012254
Davidov (2022)	1.046091	.99189013	1.1032536
Harberts (2022)	1.0443435	.99033296	1.1012995
Marion (2021)	1.1633987	1.0498273	1.2892566
Rabinowich (2021)	1.0541186	.99876535	1.1125396
Raszeja-Wyszomirska (2022)	1.0441229	.98984092	1.1013817
Toniutto (2022)	1.0456557	.98742944	1.1073153
Cuadrado (2022)	1.0484689	.99392259	1.1060088
D’Offizi (2021)	1.0376058	.98341727	1.0947801
Guarino (2022)	1.0537735	.99897957	1.1115729
Herrera (2021)	1.0446959	.99080735	1.1015152
Meunier (2022)	1.0417205	.98766047	1.0987394
Rabinowich (2021)	1.0432321	.98891342	1.1005343
Raszeja-Wyszomirska (2022)	1.0421345	.98823255	1.0989764
Toniutto (2022)	1.0527692	.99848503	1.1100045
Cholankeril (2021)	1.0523456	.99432749	1.1137491
D’Offizi (2021)	1.0430151	.98884875	1.1001484
Giannella (2022)	1.0366259	.98383445	1.09225
Guarino (2022)	1.045532	.99134195	1.1026841
Marion (2021)	1.1718735	1.0549396	1.3017687
Ericka (2022)	1.0432417	.98972487	1.0996524
Combined	1.048469	.99392261	1.1060088

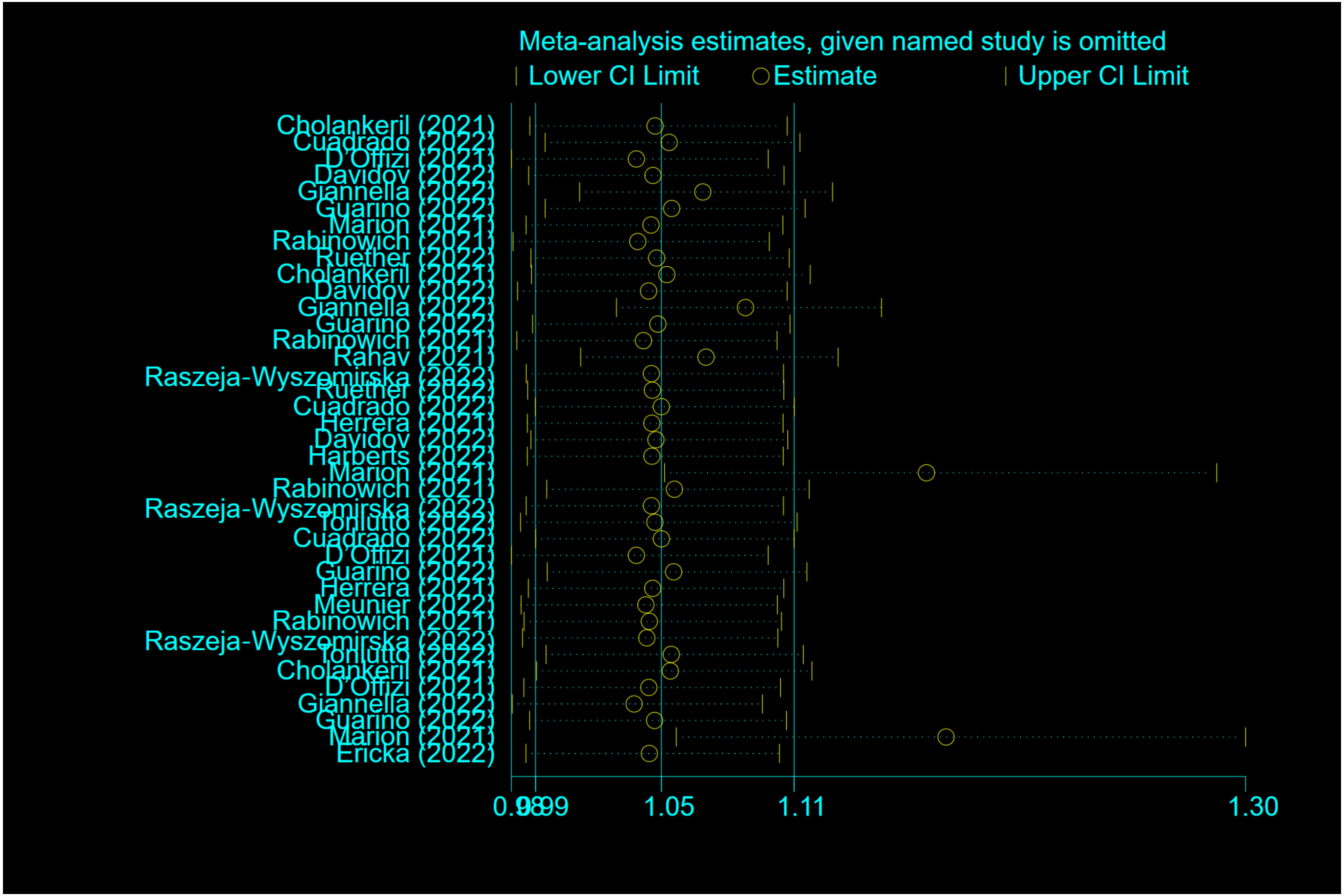

Meta-analysis estimates, given named study is omitted
Lower CI Limit
Estimate
Upper CI Limit
Cholankeril (2021)
Cuadrado (2022)
D'Offizi (2021)
Davidov (2022)
Giannella (2022)
Guarino (2022)
Marion (2021)
Rabinowich (2021)
Ruether (2022)
Cholankeril (2021)
Davidov (2022)
Giannella (2022)
Guarino (2022)
Rabinowich (2021)
Rahav (2021)
Raszeja-Wyszomirska (2022)
Ruether (2022)
Cuadrado (2022)
Herrera (2021)
Davidov (2022)
Harberts (2022)
Marion (2021)
Rabinowich (2021)
Raszeja-Wyszomirska (2022)
Toniutto (2022)
Cuadrado (2022)
D'Offizi (2021)
Guarino (2022)
Herrera (2021)
Meunier (2022)
Rabinowich (2021)
Raszeja-Wyszomirska (2022)
Toniutto (2022)
Cholankeril (2021)
D'Offizi (2021)
Giannella (2022)
Guarino (2022)
Marion (2021)
Ericka (2022)
1.05
1.11
1.30
