# Supplementary material for: Factors influencing immunogenicity and safety of SARS-CoV-2 vaccine in liver transplantation recipients: a systematic review and meta-analysis
Source: Front Immunol. 2023 Sep 5;14:1145081. doi: 10.3389/fimmu.2023.1145081 (PMC10508849; doi:10.3389/fimmu.2023.1145081)
Supplement: Supplementary file 9 [file DataSheet_6.pdf]

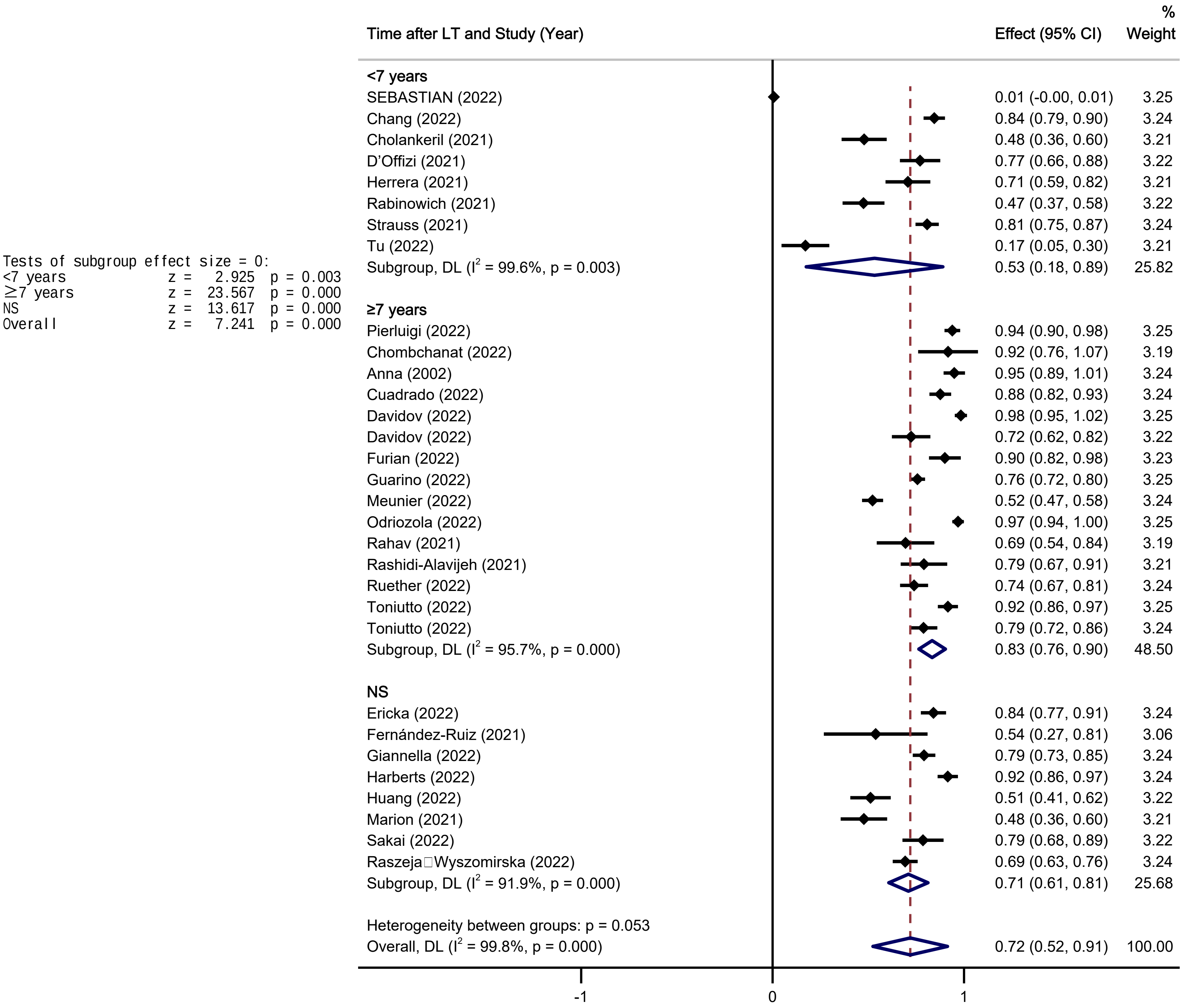

NOTE: Weights and between-subgroup heterogeneity test are from random-effects model

Study omitted	Estimate	[95% Conf. Interval]	
SEBASTIAN (2022)	.61211258	.45044485	.77378023
Herrera (2021)	.5078401	.1246037	.89107651
Rabinowich (2021)	.54081672	.14851947	.93311405
Micaela (2022)	.53261137	.17570868	.88951403
Chang (2022)	.48767105	.13478328	.84055883
John (2022)	.53261137	.17570868	.88951403
D'Offizi (2021)	.49870551	.1183308	.87908024
Strauss (2021)	.49309066	.12475786	.86142343
Tu (2022)	.58386052	.18992442	.97779661
Cholankeril (2021)	.54033631	.14974543	.93092722
Combined	.53261135	.17570868	.88951402

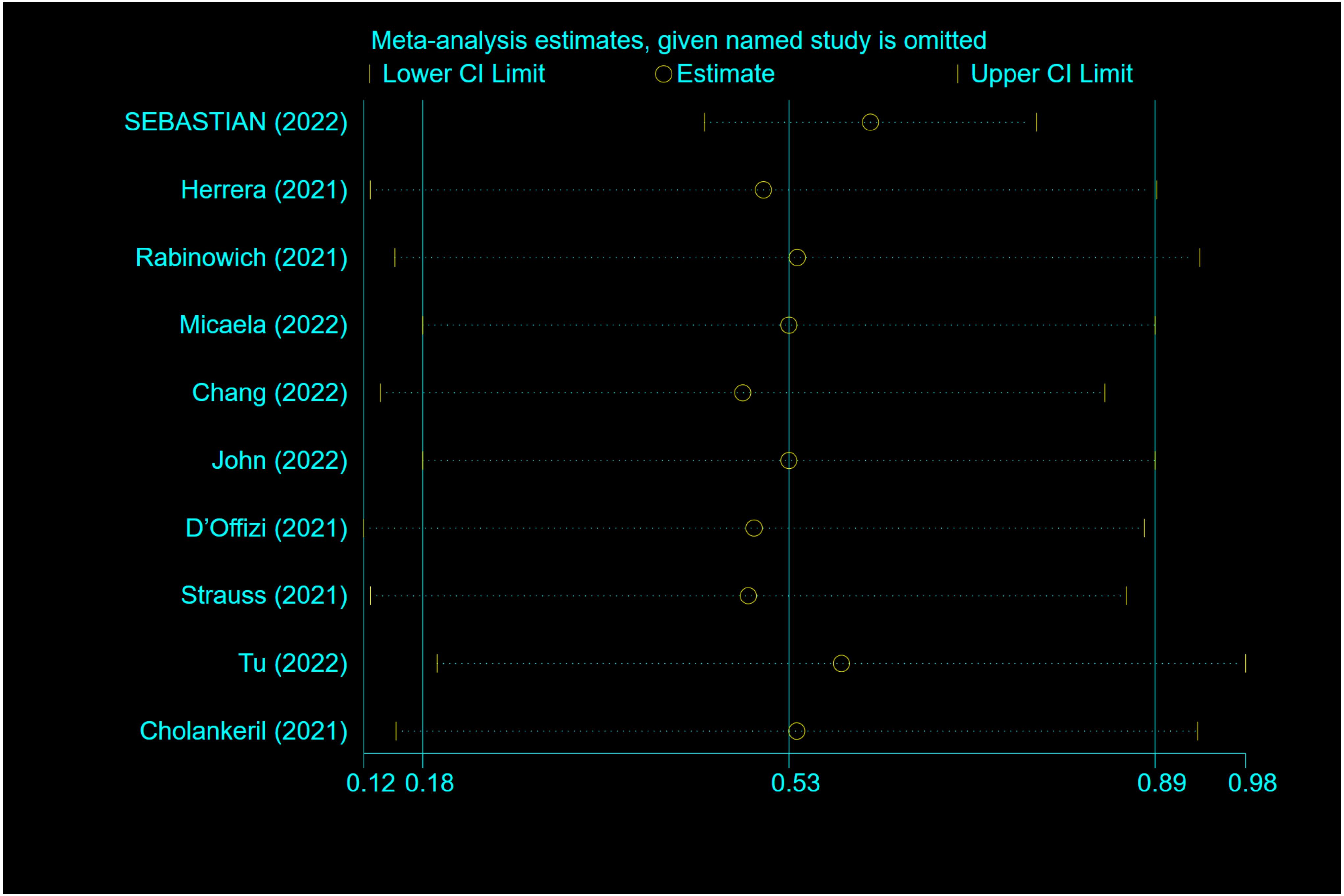

Meta-analysis estimates, given named study is omitted
Lower CI Limit
Estimate
Upper CI Limit
SEBASTIAN (2022)
Herrera (2021)
Rabinowich (2021)
Micaela (2022)
Chang (2022)
John (2022)
D'Offizi (2021)
Strauss (2021)
Tu (2022)
Cholankeril (2021)
0.12
0.18
0.53
0.89
0.98

Study omitted	Estimate	[95% Conf.	Interval]
Pierluigi (2022)	.82519609	.74953192	.90086019
Chombchanat (2022)	.82878208	.75713241	.90043169
Anna (2002)	.82470119	.75090331	.89849901
Cuadrado (2022)	.83015388	.7557748	.90453303
Davidov (2022)	.82176906	.74825239	.89528579
Davidov (2022)	.84095991	.76948369	.91243613
Furian (2022)	.82870144	.75575578	.90164703
Guarino (2022)	.8394168	.76745087	.91138268
Meunier (2022)	.86019641	.8081252	.91226763
Odriozola (2022)	.8228057	.74765778	.89795357
Rahav (2021)	.84152162	.77041882	.91262442
Rashidi-Alavijeh (2021)	.83620435	.76431161	.90809715
Ruether (2022)	.84036702	.76864314	.91209096
Toniutto (2022)	.82711208	.75250179	.90172237
Toniutto (2022)	.83671021	.76401889	.9094016
Combined	.83350907	.76418871	.90282943

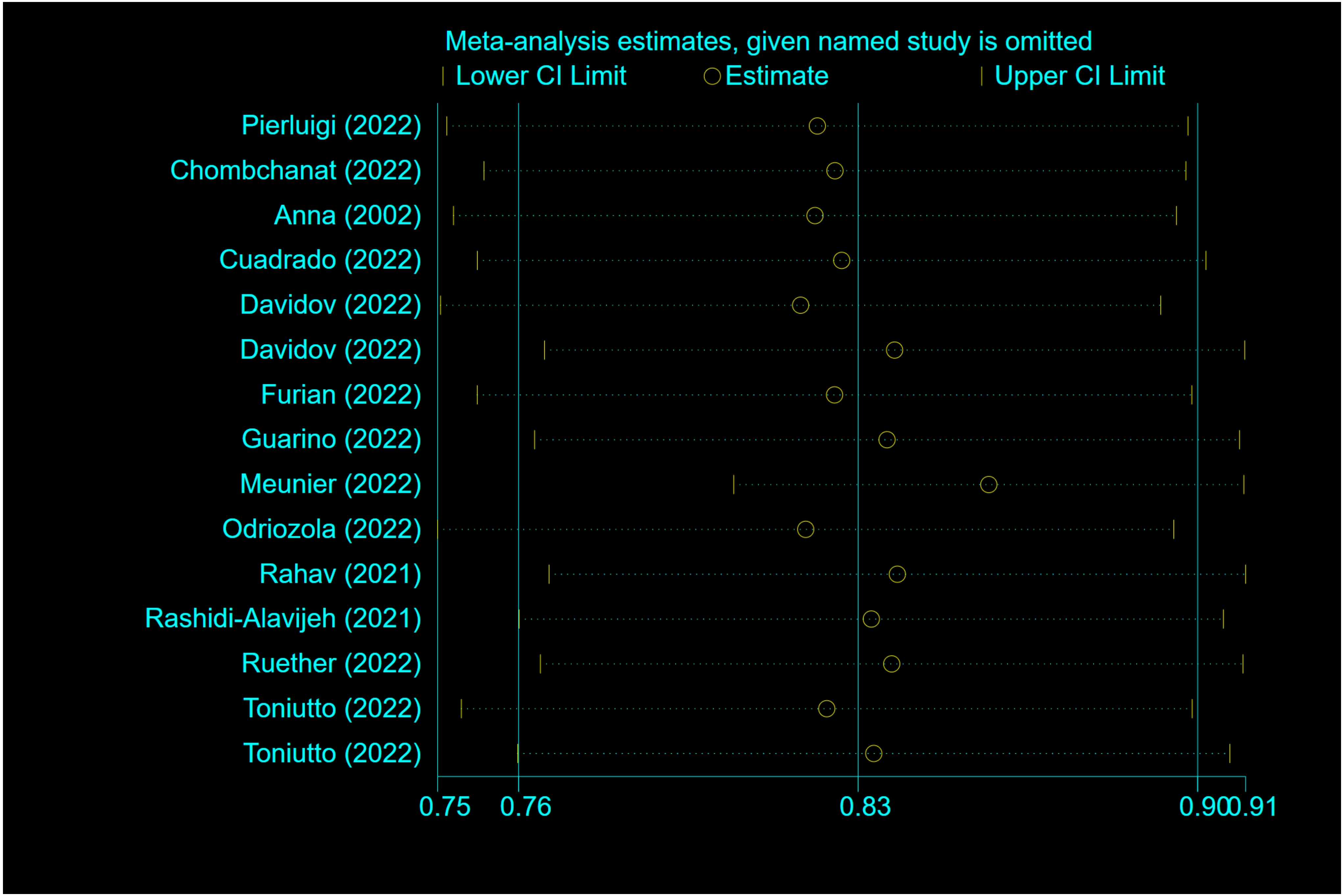
Meta-analysis estimates, given named study is omitted
Lower CI Limit
Estimate
Upper CI Limit
Pierluigi (2022)
Chombchanat (2022)
Anna (2002)
Cuadrado (2022)
Davidov (2022)
Davidov (2022)
Furian (2022)
Guarino (2022)
Meunier (2022)
Odriozola (2022)
Rahav (2021)
Rashidi-Alavijeh (2021)
Ruether (2022)
Toniutto (2022)
Toniutto (2022)
0.75
0.76
0.83
0.900.91
