# Supplementary material for: Factors influencing immunogenicity and safety of SARS-CoV-2 vaccine in liver transplantation recipients: a systematic review and meta-analysis
Source: Front Immunol. 2023 Sep 5;14:1145081. doi: 10.3389/fimmu.2023.1145081 (PMC10508849; doi:10.3389/fimmu.2023.1145081)
Supplement: Supplementary file 10 [file DataSheet_7.pdf]

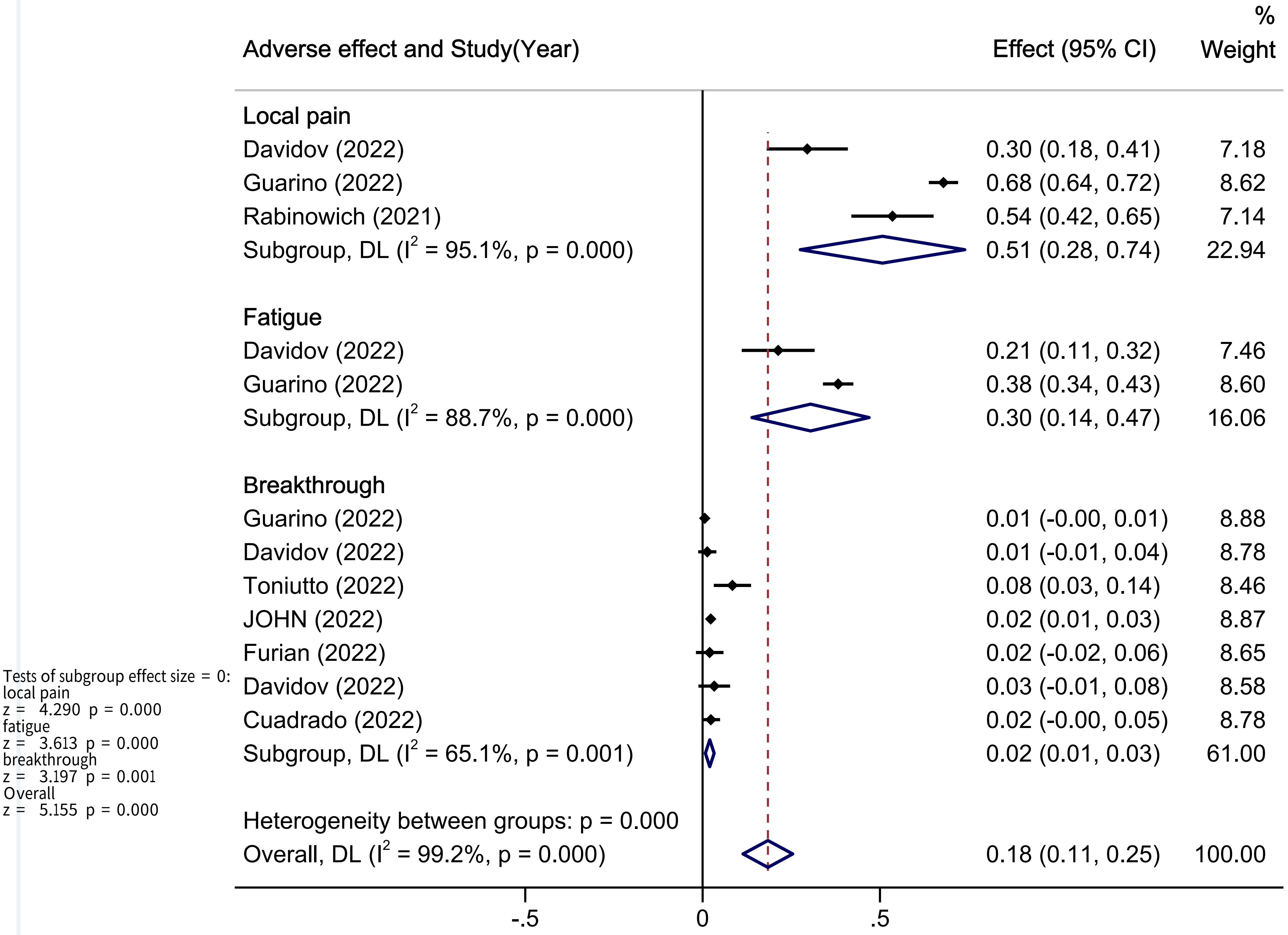

NOTE: Weights and between-subgroup heterogeneity test are from random-effects model

Study omitted	Estimate	[95%	Conf. Interval]
Davidov (2022)	.17547378	.10315842	.24778913
Davidov (2022)	.18176459	.1090884	.25444078
Guarino (2022)	.12157243	.07902378	.16412109
Guarino (2022)	.1639135	.09665228	.23117474
Rabinowich (2021)	.15693614	.08599771	.22787456
Guarino (2022)	.20560446	.09517492	.31603402
Davidov (2022)	.20116264	.1247809	.27754438
Toniutto (2022)	.19350453	.11969706	.26731199
JOHN (2022)	.20406184	.09295754	.31516612
Furian (2022)	.19993356	.12534989	.27451721
Davidov (2022)	.1985289	.12429618	.27276161
Cuadrado (2022)	.20017894	.12385411	.27650374
Combined	.18412226	.11411263	.2541319

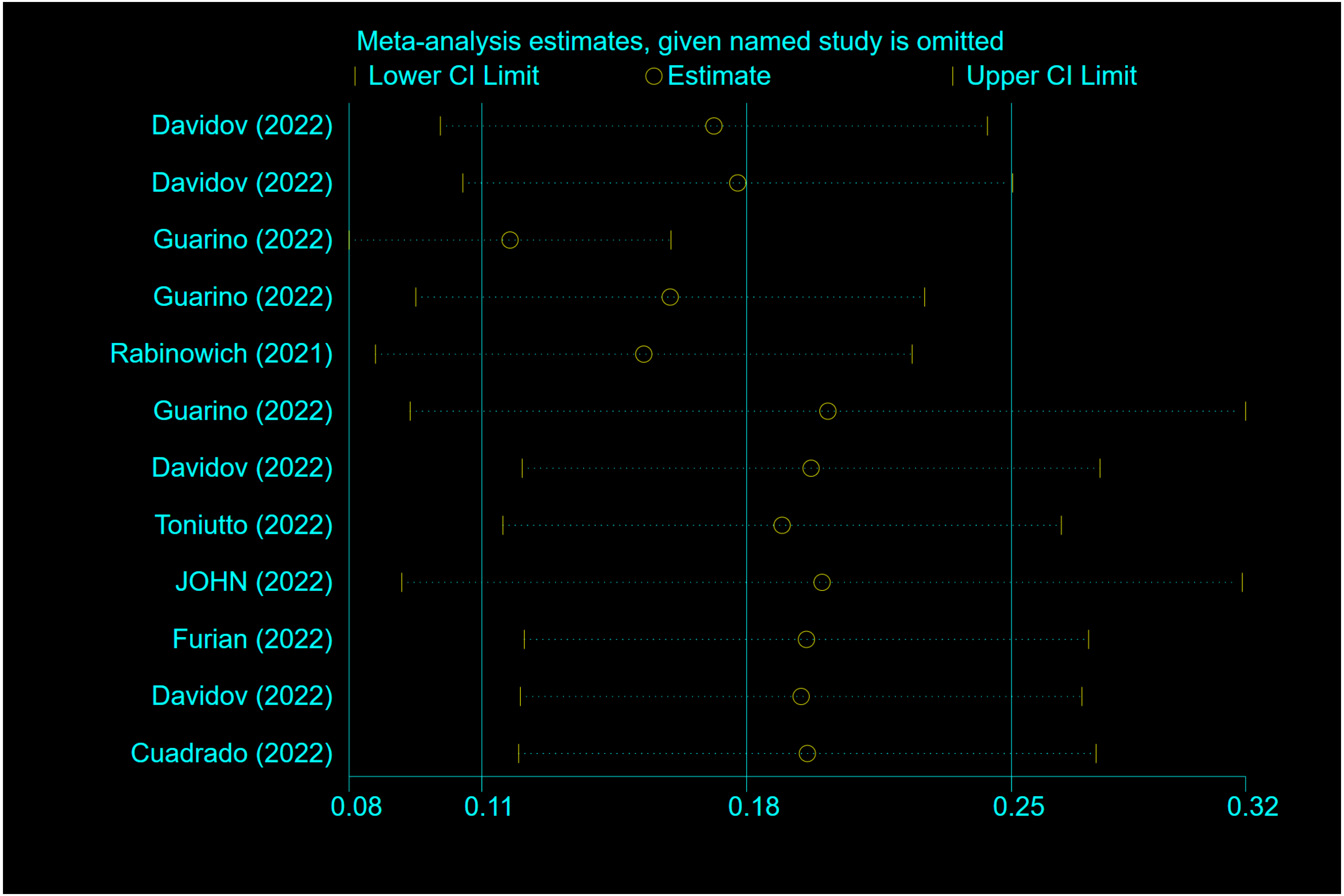
Meta-analysis estimates, given named study is omitted
Lower CI Limit
Estimate
Upper CI Limit
Davidov (2022)
Davidov (2022)
Guarino (2022)
Guarino (2022)
Rabinowich (2021)
Guarino (2022)
Davidov (2022)
Toniutto (2022)
JOHN (2022)
Furian (2022)
Davidov (2022)
Cuadrado (2022)
0.08
0.11
0.18
0.25
0.32
